# A comparison of different practical indices for assessing carbohydrate quality among carbohydrate-rich processed products in the US

**DOI:** 10.1371/journal.pone.0231572

**Published:** 2020-05-21

**Authors:** Junxiu Liu, Colin D. Rehm, Peilin Shi, Nicola M. McKeown, Dariush Mozaffarian, Renata Micha

**Affiliations:** 1 Friedman School of Nutrition Science and Policy, Tufts University, Boston, Massachusetts, United States of America; 2 Department of Epidemiology and Population Health, Albert Einstein College of Medicine, Bronx, New York, United States of America; 3 Jean Mayer USDA Human Nutrition Research Center on Aging at Tufts University, Boston, Massachusetts, United States of America; Higher Institute of Applied Sciences and Technology of Gabes University of Gabes, TUNISIA

## Abstract

Healthier carbohydrate (carb)-rich foods are essential for health, but practical, validated indices for their identification are not established. We compared four pragmatic metrics, based on, per 10g of carb:(a) ≥1g fiber (10:1 carb:fiber), (b) ≥1g fiber and <1g free sugars (10:1:1 carb:fiber:free sugars), (c) ≥1g fiber and <2g free sugars (10:1:2 carb:fiber:free sugars); and (d) ≥1g fiber and, per each 1 g of fiber, <2g free sugars (10:1 carb:fiber, 1:2 fiber:free sugars; or 10:1|1:2). Using 2013–2016 National Health and Nutrition Examination Survey /Food and Nutrient Database for Dietary Studies, we assessed, overall and for 12 food categories, whether each metric discriminated carb-rich products higher or lower (per 100g) in calories, total fat, saturated fat, protein, sugar, fiber, sodium, potassium, magnesium, folate, and 8 vitamins/minerals. Among 2,208 carb-rich products, more met 10:1 (23.2%) and 10:1|1:2 (21.3%), followed by 10:1:2 (19.2%) and 10:1:1 (16.4%) ratios, with variation by product sub-categories. The 10:1 and 10:1|1:2 ratios similarly identified products with lower calories, fat, free sugars, and sodium; and higher protein, fiber, potassium, magnesium, iron, vitamin B6, vitamin E, zinc and iron. The 10:1:2 and 10:1:1 ratios identified products with even larger differences in calories and free sugars, but smaller differences in other nutrients above and lower folate, thiamine, riboflavin, and niacin; the latter findings were attenuated after excluding breakfast cereals (~9% of products). These novel findings inform dietary guidance for consumers, policy, and industry to identify and promote the development of the healthier carb-rich foods.

## Introduction

Carbohydrate (carb)-rich products comprise the majority of foods in the diet. Carb-containing foods that are minimally processed–for instance, legumes, fruits, and vegetables–are generally recognized by consumers as healthful and are recommended by dietary guidelines [1]. In contrast, consumers are often confused about selecting between other, packaged carb-rich products: for example, different types of breads, breakfast cereals, salty snacks, crackers, energy/snack bars, and bakery products [2,3]. These foods, which together represent the majority of carbohydrate intake in most nations, contain varying proportions of diverse whole grains, refined grains, natural sugars, added sugars, and dietary fiber. Thus, while U.S. and other dietary guidelines recommend that the majority of grain based foods be consumed as “whole grains” [1,4], in practice, the available products include diverse combinations of different types of grains as well as sugars, making it challenging to identify and select healthier products. For example, USDA recommended definitions to identify healthier whole grain products require complex reviews of the ingredients list [5]; while an industry-sponsored “whole grain stamp” identifies products with higher fiber but these products may be high in calories and contain added sugars [6]. Other types of package marketing claims, such as “made with whole grains” or “multi-grain,” further contribute to consumer confusion. New mandatory labeling of added sugars content on the Nutrition Facts label provides additional information to the public, but does not provide information on whole grain content nor distinguish products high in refined grains and starch which may have similar metabolic harms as added sugars [7].

Clearly, practical, validated metrics are needed to help consumers identify and guide the food industry to develop and promote healthier carbohydrate products. A recent convening by the U.S. Interagency Committee on Human Nutrition Research concluded that definitions of healthier grain foods are not established and are urgently needed [8]. As one potential metric, the American Heart Association (AHA) has recommended choosing carb-rich foods with at least 1 g of fiber for every 10 g of total carbohydrate (carb:fiber ratio <10:1), based on the ratio of carbohydrate-to-fiber in whole wheat [9, 10]. This ratio aims to incorporate the overall relative amounts of starch and sugars vs. whole grains, bran, and other sources of fiber (e.g. added seeds). Researchers have found that the 10:1 ratio is more effective at identifying cereals-based products with better nutritional quality than other approaches that utilize the ingredients [6,11].

While the 10:1 ratio appears to be a valid, practical metric for identifying healthier carb-rich foods, several questions remain. First, prior studies have evaluated available products in major supermarkets, rather than actual national intakes of products. Second, the 10:1 ratio explicitly does not differentiate contents of starch vs. sugar. With the growing emphasis on reducing added or free sugars [1, 12, 13], it is unclear whether modified ratios with additional restrictions on free sugar content may improve performance and/or limit available product choices for consumers. Given the public health impact of carbohydrate quality, it is crucial to test practical metrics for identifying carb-rich products with higher nutritional quality.

To address these gaps in the knowledge, we investigated how four different metrics, including the 10:1 ratio and three additional metrics further limiting free sugars, related to the nutritional quality of carb-rich products in the US.

## Materials and methods

### Metrics of carbohydrate quality

We evaluated four metrics of carbohydrate quality based on the ratios of total carbohydrate, fiber, and/or free sugars. These metrics were selected as pragmatic in that they are widely measured, based on nutrients commonly listed on product labels, and more straightforward than complex nutrition profiling scoring schemes. The first was the original validated 10:1 carb:fiber ratio (10:1),[14–17] defined as ≥1 g of fiber per 10 g of carbohydrate. We evaluated three additional metrics incorporating additional restrictions on free sugars contents, per 10g of carb, including:

- ≥1 g of fiber *and* < 1 g of free sugars (10:1:1 ratio of carb:fiber:free sugars, or 10:1:1);
- ≥ 1 g of fiber *and* < 2 g of free sugars (10:1:2 ratio of carb:fiber:free sugars, or 10:1:2); and
- ≥1 g of fiber *and*, per each 1 g of fiber, <2g free sugars (10:1 ratio of carb:fiber + 1:2 ratio of fiber:free sugars; or 10:1|1:2).

The 10:1:1 was selected based on the recommendation of consumption of 50% of energy from carbohydrates, 5% of energy from free sugars and 30g of fiber [18,19]. The 10:1:2 was similarly selected, but with the more permissive assumption that the 10% energy limit on added sugars would largely be derived from these carb-rich foods [20]. The 10:1|1:2 was selected to limit the amount of free sugars depending on the fiber (rather than carbohydrate) content of the product, based on the WHO recommendations to consume 30 g/d of fiber and no more than 10% energy (60 g on a 2400 kcal/d diet) from free sugars [21].

### Identification of carb-rich products consumed in the US

We combined data from the two most recent versions of the Food and Nutrient Database for Dietary Studies (FNDDS, 2013–2014 and 2015–2016) [22], corresponding to the National Health and Nutrition Examination Survey (NHANES) for this period. These data were supplemented with information from the Food Patterns Equivalents Database (FPED) [23], which includes the amounts of grains and other food components in these products. Information on free sugars content was adapted from the UK definition [24] for application in FNDDS using added sugars (e.g., honey, white sugars, syrups), sugars from fruit juice, other sugars present in beverages (excluding natural dairy sugars), and sugars from extruded fruit/vegetable products. We selected categories of carb-rich products for which metrics of nutritional quality would be most relevant to consumers and industry, based on the What We Eat in America (WWEIA) food classification developed by the United States Department of Agriculture (USDA) [25]. We selected 12 total categories, including breads, rolls, and tortillas; quick breads; ready-to-eat (cold) cereals; cooked cereals; cooked grains; sweet bakery products; savory snacks; crackers; snack/meal bars; smoothies and grain drinks; baby food: cereals, snacks, sweets; and relevant mixed dishes (e.g., rice/pasta mixed dishes). We focused on these variably processed foods, and did not include unprocessed foods (e.g., fruits, vegetables, legumes). The flowchart of the numbers of food items included is shown in S1 Fig.

We evaluated the identified products in these categories both overall and weighted by the frequency of consumption among US adults and children from the two most recent corresponding cycles of NHANES (2013–2014, 2015–2016). Nationally weighted analyses were considered overall and separately for adults age 20+ years and children age 2–19 years.

### Nutritional profiles

To assess the nutritional profiles of each product, we utilized FNDDS data to quantify the contents of calories, total fat, saturated fat, protein, total sugar, free sugars, added sugar, fiber, sodium, potassium, magnesium, folate, thiamine, riboflavin, niacin, vitamin B6, vitamin B12, vitamin E, zinc and iron. All nutrients were selected *a priori* based on those commonly contained in whole grains or fortified in grain-rich foods. To account for varying serving sizes of different products, all analyses were standardized to 100g servings.

In addition to the individual nutrients above, we also evaluated the extent to which each product met selected nutrient profiling systems (NPS) currently used in practice, including from the UK Ofcom (the Office of Communications) [26], FSANZ (Food Standards Australia New Zealand) [27] and the Health Star Rating (Australia New Zealand) [28]. We recognized that these nutrient profiling systems do not capture all aspects of nutritional quality, in particular higher contents of many healthful compounds. Yet, these systems are currently being used by governments, for example Ofcom and FSANZ Nutrient Profiling Scoring Criterion (NPSC) to determine if a product can carry a particular ‘health claim’ [29,30]; and the Health Star Rating (derived from NPSC with a slightly differing algorithm) for front-of-pack labeling for packaged foods [31]. In brief, foods were first classified into specific categories with two for the Ofcom model, three for the FSANZ NPSC and six for the HSR. Then, a summary score was calculated for each food product based on points for both nutrients to limit (energy, saturated fat, total sugar, and sodium) and nutrients or food components to encourage (protein, fiber, and percent composition of fruits, vegetables, nuts and legumes (FVNLs) in a product). Points are allocated based on the nutritional content in 100g of a food or drink. The details of components and scoring of these three nutrient profiling systems are presented in the Supplemental Material.

### Statistical analysis

For all eligible carb-rich products, we assessed the numbers and proportions of products meeting each metric, both overall (all available products) and weighted by their consumption levels in the U.S. Kappa statistics were calculated to assess chance-corrected agreement between the metrics.

Our primary outcomes were the nutritional differences according to each metric for all carb-rich foods combined, which can be considered the mean differences in nutrient contents if consumers were to make selections based on these metrics among different carb-rich products. The dependent variables were each of the proposed nutrients in nutritional profile. The independent variable was a dichotomous indicator of products meeting or not meeting each ratio. We used robust standard error of variance in the regressions to account for non-normally distributed nutrients [32]. In secondary analyses, we repeated the analyses among each of the 12 food categories, which can be considered the mean differences in nutrient contents if consumers were to make selections based on these metrics within specific food categories. To assess and compare the proportion meeting each of the three NPS thresholds, we used similar approaches with logistic regression.

A 2-tailed P value < .05 was taken to indicate statistical significance. All statistical analyses were conducted in Stata software, version 14.2 (StatCorp LLC, College Station, Texas), and R, version 3.4.2 (R Foundation for Statistical Computing).

## Results

### Carb-rich products available in the U.S. diet

A total of 2,208 available carb-rich products were identified in FNDDS, including 386 (17.5%) sweet bakery products; 206 (9.3%) breads, rolls, and tortillas; 198 (9.0%) cold cereals; 197 (8.9%) cooked cereals; 177 (8.0%) savory snacks; 143 (6.5%) quick breads and bread products; 82 (3.7%) cooked grains; 80 (3.6%) crackers; 46 (2.1%) snack/meal bars; 19 (0.86%) smoothies and grain drinks; 55 (2.5%) baby foods; and 619 (28.0%) mixed dishes (Table 1).

**Table 1:** Comparison of carbohydrate-rich products in the U.S. meeting each of four proposed metrics for assessing carbohydrate quality^a^.

Food Categories^b^	No. of Products	Percent Meeting each Metric^c^				Kappa^d^
		10:1	10:1:1	10:1:2	10:1|1:2	
Sweet bakery products	386	2.6	0.8	0.8	1.3	0.63
		(1.0–4.2)	(0.0–1.7)	(0.0–1.7)	(0.2–2.4)	
Bread, rolls, tortillas	206	31.1	24.8	31.1	31.1	0.92
		(24.7–37.4)	(18.8–30.7)	(24.7–37.4)	(24.7–37.4)	
Cold cereals	198	43.9	9.1	20.7	34.3	0.50
		(37.0–50.9)	(5.1–13.1)	(15.0–26.4)	(27.7–41.0)	
Cooked cereals	197	38.6	29.9	33.5	38.1	0.89
		(31.8–45.4)	(23.5–36.4)	(26.9–40.1)	(31.3–44.9)	
Savory snacks	177	26.0	24.9	24.9	25.4	0.98
		(19.5–32.5)	(18.5–31.2)	(18.5–31.2)	(19.0–31.9)	
Cooked grains	82	32.9	32.9	32.9	32.9	1.0
		(22.7–43.2)	(22.7–43.2)	(22.7–43.2)	(22.7–43.2)	
Crackers	80	32.5	21.3	31.3	31.3	0.86
		(22.2–42.8)	(12.2–30.3)	(21.0–41.5)	(21.0–41.5)	
Snack/meal bars	46	23.9	0	0	13.0	0.16
		(11.4–36.4)			(3.2–22.9)	
Smoothies and grain drinks	19	42.1	0	0	0	0
		(19.3–64.9)				
Baby food:cereals, snacks, sweets	55	7.3	5.5	5.5	5.5	0.92
		(0.3–14.2)	(0–11.5)	(0–11.5)	(0–11.5)	
Mixed dishes	619	23.1	21.8	22.8	23.1	0.98
		(19.8–26.4)	(18.6–25.1)	(19.5–26.1)	(19.8–26.4)	
**Overall**	2208	23.2	16.4	19.2	21.3	0.88
		(21.4–25.0)	(14.8–17.9)	(17.5–20.8)	(19.6–23.0)	

^a^ We used data from the two most recent National Health and Nutrition Examination Survey (NHANES) cycles (2013–14, 2015–16) to identify carbohydrate (carb)-rich products in the US diet.

^b^ Products were aggregated into 12 food categories based on the WWEIA food categories. The mixed dish category included rice mixed dishes, pasta mixed dishes, macaroni and cheese, turnovers and other grain-based items, fried-risk and lo/chow mein. Additional mixed dishes including (e.g., meat mixed dishes, sandwiches, soups, pizza, burgers, etc.) were included if they contained ≥ 50% of energy from carbohydrates and ≥0.25 ounce-equivalents of total grains.

^c^ We defined and applied four different carb ratios based on, per 10g of carb:(a) ≥1g fiber (10:1 carb:fiber), (b) ≥1g fiber and <1g free sugars (10:1:1 carb:fiber:free sugars), (c) ≥1g fiber and <2g free sugars (10:1:2 carb:fiber:free sugars); and (d) ≥1g fiber and, per each 1 g of fiber, <2g free sugars (10:1 carb:fiber, 1:2 fiber:free sugars; or 10:1|1:2). Values represent number (#) of products and unweighted percent (%) of all available food products (consumed, not consumed) meeting each of the ratios.

^d^Kappa is calculated based on the difference between how much agreement is actually present compared to how much agreement would be expected to be present by chance alone. 0 indicates the agreement is by chance; 0.01–0.20 indicates slight agreement; 0.21–0.40 indicates fair agreement; 0.41–0.60 indicates moderate agreement; 0.61–0.80 indicates substantial agreement; 0.81–0.99 indicates almost perfect agreement.

### Proportions of food products meeting each proposed metric

Among these food products, more than 3 in 4 (>76.8%) did not meet any of the metrics. The highest number met the 10:1 (23.2%), followed by the 10:1|1:2 (21.3%), 10:1:2 (19.2%), and 10:1:1 (16.4%) (overall agreement across metrics [kappa] = 0.88) (Table 1). Considerable variation was identified across the different food categories. For example, 43.9% of cold cereals, 38.6% of cooked cereals, and 31.1% of breads, but only 2.6% of sweet bakery products, met the 10:1. Agreement across the metrics was also highly variable by food category. For example, agreement was very high (kappa> = 0.98) for cooked grains, savory snacks, and mixed dishes. Agreement was lowest (kappa< = 0.50) for cold cereals, snack/meal bars, and smoothies/grain drinks. Generally, the 10:1 and 10:1|1:2 identified a more similar proportion of products meeting their criteria, except for cold cereals (43.9 vs. 34.3%, respectively), snack/meal bars (23.9 vs. 13.0%), and smoothies/grain drinks (42.1 vs. 0%). The 10:1:1 was most restrictive, with largest differences compared to other metrics in products identified for cold cereals (e.g., only 9.1% met 10:1:1, vs. 20.7% for 10:1:2), crackers (21.3 vs. 31.3%), and breads (24.8 vs. 31.1%). Across all products, intercorrelations between the four metrics ranged from 0.79 for 10:1 and 10:1:1 to 0.95 for 10:1 and 10:1|1:2 (S1 Table in S1 File).

Weighted by the frequency consumption, the general patterns across metrics and food categories were similar (Table 2). However, the overall percentages of products meeting each metric were lower when weighted by consumption as compared to available choices, indicating that products not meeting the metrics were more frequently consumed. For example, while 16.4% of available products met the 10:1:1 ratio (Table 1), only 9.7% of products met this metric when weighted by actual consumption levels. The percentage of food products meeting each metric was also generally lower among foods consumed by children as compared with those consumed by adults. For example, overall, 15.5% of products consumed by children vs. 19.7% of products consumed by adults met the 10:1 ratio.

**Table 2:** Comparison of consumed carbohydrate-rich products meeting each of four proposed metrics for assessing carbohydrate quality by Americans^a^.

Food Categories^b^	Analyses^c^	Products^d^		% Meeting each Metric^e^			
		#	Frequency (million)	10:1	10:1:1	10:1:2	10:1|1:2
**Sweet bakery products**	*Overall*	313	538.1	0.3	0.1	0.1	0.2
				(0.0, 0.5)	(0.0, 0.2)	(0.0, 0.2)	(0.0, 0.4)
	*Adults*	303	396.4	0.3	0.1	0.1	0.2
				(0.0, 0.6)	(0.0, 0.3)	(0.0, 0.3)	(0.0, 0.5)
	*Children*	226	141.7	0.1	0	0	0
				(0.0, 0.3)			
	*Children*	80	12.3	60.5	41.4	44.2	60.5
				(40.6, 80.5)	(20.2, 62.6)	(22.8, 65.5)	(40.6, 80.5)
**Savory snacks**	*Overall*	150	447.7	16.7	16.6	16.6	16.7
				(7.2, 26.3)	(7.1, 26.2)	(7.1, 26.2)	(7.1, 26.2)
	*Adults*	144	308.9	16.7	16.6	16.6	16.6
				(7.1, 26.2)	(7.0, 26.1)	(7.0, 26.1)	(7.1, 26.2)
	*Children*	136	138.8	16.8	16.7	16.7	16.7
				(7.0, 26.6)	(7.0, 26.5)	(7.0, 26.5)	(7.0, 26.5)
	*Children*	95	57.9	3.2	1.7	3.1	3.2
				(0.0, 6.5)	(0.0, 3.7)	(0.0, 6.3)	(0.0, 6.5)
	*Adults*	70	149.2	7.2	7.2	7.2	7.2
				(0.0, 14.4)	(0.0, 14.4)	(0.0, 14.4)	(0.0, 14.4)
	*Adults*	70	154.0	16.3	5.3	15.1	15.1
				(2.9, 29.6)	(0.6, 10.1)	(2.1, 28.1)	(2.1, 28.1)
	*Children*	54	49.3	11.7	6.5	11.5	11.5
				(0.0, 24.0)	(0.0, 16.7)	(0.0, 23.7)	(0.0, 23.7)
**Snack/meal bars**	*Overall*	44	92.6	20.9	0	0	10.9
				(4.1, 37.6)			(0.0, 24.0)
	*Adults*	42	70.8	21.2	0	0	11.2
				(4.1, 38.3)			(0.0, 24.1)
	*Children*	38	21.8	19.9	0	0	10.0
				(1.8, 38.0)			(0.0, 24.4)
**Smoothies and grain drinks**	*Overall*	19	42.7	76.6	0	0	0
				(55.8, 97.5)			
	*Adults*	19	35.3	79.9	0	0	0
				(61.4, 98.4)			
	*Children*	18	7.3	60.9	0	0	0
				(28.1, 93.6)			
	*Adults*	2	0.03	0	0	0	0
	*Children*	13	1.00	1.9	1.9	1.9	1.9
				(0.0, 6.0)	(0.0, 6.0)	(0.0, 6.0)	(0.0, 6.0)
**Mixed dishes**	*Overall*	471	422.8	13.7	12.1	13.6	13.7
				(8.4, 19.0)	(7.1, 17.2)	(8.3, 18.9)	(8.4, 19.0)
	*Adults*	425	305.5	13.3	12.8	13.3	13.3
				(8.0, 18.6)	(7.6, 18.0)	(8.0, 18.6)	(8.0, 18.6)
	*Children*	339	117.3	14.6	10.4	14.4	14.6
				(7.5, 21.6)	(5.2, 15.5)	(7.4, 21.5)	(7.5, 21.6)
**All products**	*Overall*	1736	3312.0	18.6	9.7	14.8	16.5
				(13.5, 23.7)	(6.5, 12.8)	(10.0, 19.7)	(11.5, 21.5)
	*Adults*	1621	2449.4	19.7	10.2	15.9	17.7
				(14.2, 25.1)	(6.9, 13.5)	(10.7, 21.1)	(12.4, 23.0)
	*Children*	1330	862.6	15.5	8.1	12.0	13.1
				(10.7, 20.3)	(5.0, 11.1)	(7.7, 16.2)	(8.7, 17.4)

^a^ We used data from the two most recent National Health and Nutrition Examination Survey (NHANES) cycles (2013–14, 2015–16) to identify carbohydrate (carb)-rich products consumed by Americans.

^b^ Products were aggregated into 12 food categories based on the WWEIA food categories. The mixed dish category included rice mixed dishes, pasta mixed dishes, macaroni and cheese, turnovers and other grain-based items, fried-risk and lo/chow mein. Additional mixed dishes including (e.g., meat mixed dishes, sandwiches, soups, pizza, burgers, etc.) were included if they contained ≥ 50% of energy from carbohydrates and ≥0.25 ounce-equivalents of total grains.

^c^ Children included individuals aged 2–19 years old and adults included individuals aged 20 years and older.

^d^Frequency means that products were weighted by their actual reported consumption levels overall or by children or adults, with products consumed more frequently receiving greater weight.

^e^ We defined and applied four different carb ratios based on, per 10g of carb:(a) ≥1g fiber (10:1 carb:fiber), (b) ≥1g fiber and <1g free sugars (10:1:1 carb:fiber:free sugars), (c) ≥1g fiber and <2g free sugars (10:1:2 carb:fiber:free sugars); and (d) ≥1g fiber and, per each 1 g of fiber, <2g free sugars (10:1 carb:fiber, 1:2 fiber:free sugars; or 10:1|1:2).

### Nutritional quality of food products meeting or not meeting each proposed metric

When we assessed the nutrient contents of products meeting or not meeting each proposed metric, all metrics each identified products with significantly lower calories, fat, and sugars, and higher protein, fiber, potassium, and magnesium. The 10:1 and 10:1|1:2 metrics each could further identify products with higher vitamin B6, vitamin E, zinc, and iron. The 10:1, 10:1:1 and 10:1|1:2 metrics each could identify products with lower sodium. The 10:1 metric is the only one, which can identify higher values of vitamin B12 (Table 3). Compared to the 10:1 and 10:1|1:2 ratios, the 10:1:2 and 10:1:1 metrics identified larger differences in contents of calories and sugars, but smaller differences in several of the other nutrients above including protein, fiber, potassium, and magnesium. In addition, the 10:1:2 and 10:1:1 ratios also identified products with significantly lower folic acid, thiamine, riboflavin, and niacin than products not meeting these ratios.

**Table 3:** Nutrient composition of available carbohydrate-rich products meeting or not meeting each of four proposed metrics for assessing carbohydrate quality in the US.

		Meeting or not Meeting each Metric^a^											
Composition^b^	Values^c^	10:1			10:1:1			10:1:2			10:1|1:2		
		Yes	No	Dif.^d^	Yes	No	Dif.^d^	Yes	No	Dif.^d^	Yes	No	Dif.^d^
	# (%) products	512 (23.2%)	1696 (76.8%)		362 (16.4%)	1846 (83.6%)		423 (19.2%)	1785 (80.8%)		471 (21.3%)	1737 (78.7%)	
**Calories (kcal)**	mean (SE)	245.4	272.9	-27.5***	221.1	275.5	-54.3***	232.8	274.6	-41.8***	240.7	273.6	-32.9***
		(6.2)	(3.2)	(7.0)	(7.5)	(3.1)	(8.1)	(6.8)	(3.2)	(7.5)	(6.4)	(3.2)	(7.2)
**Total fat (g)**	mean (SE)	7.21	9.21	-2.0***	7.54	8.98	-1.44 **	7.39	9.07	-1.68***	7.28	9.14	-1.86***
		(0.35)	(0.20)	(0.40)	(0.46)	(0.19)	(0.50)	(0.41)	(0.19)	(0.45)	(0.37)	(0.20)	(0.42)
**Saturated fat (g)**	mean (SE)	1.95	2.63	-0.68***	2.1	2.55	-0.45 **	1.99	2.59	-0.60 ***	1.92	2.63	-0.71***
		(0.14)	(0.07)	(0.15)	(0.18)	(0.07)	(0.19)	(0.15)	(0.07)	(0.17)	(0.14)	(0.07)	(0.16)
**Protein (g)**	mean (SE)	7.5	6.08	1.42***	7.09	6.27	0.82***	7.42	6.17	1.25***	7.58	6.09	1.49***
		(0.18)	(0.08)	(0.20)	(0.19)	(0.08)	(0.21)	(0.18)	(0.08)	(0.20)	(0.19)	(0.08)	(0.20)
**Total sugar (g)**^e^	mean (SE)	6.76	11.7	-4.94***	2.37	12.16	-9.79***	3.74	12.17	-8.43***	5.21	12.01	-6.8***
		(0.41)	(0.33)	(0.53)	(0.18)	(0.31)	(0.36)	(0.26)	(0.32)	(0.41)	(0.33)	(0.33)	(0.47)
**Added sugars (g)**^e^	mean (SE)	4.55	9.46	-4.91***	0.56	9.85	-9.29 ***	1.68	9.9	-8.22***	3.1	9.74	-6.65***
		(0.37)	(0.31)	(0.61)	(0.06)	(0.3)	(0.67)	(0.16)	(0.31)	(0.64)	(0.26)	(0.31)	(0.62)
**Free sugars (g)**^e^	mean (SE)	4.64	9.55	-4.9***	0.56	9.95	-9.39***	1.68	10	-8.32***	3.1	9.85	-6.75***
		(0.37)	(0.32)	(0.49)	(0.06)	(0.30)	(0.31)	(0.16)	(0.31)	(0.35)	(0.26)	(0.31)	(0.41)
**Fiber (g)**	mean (SE)	6.31	2.05	4.26 ***	5.22	2.61	2.62***	5.63	2.42	3.2***	6.23	2.17	4.06***
		(0.23)	(0.04)	(0.23)	(0.24)	(0.07)	(0.25)	(0.23)	(0.06)	(0.24)	(0.25)	(0.04)	(0.25)
**Sodium (mg)**	mean (SE)	343.2	379.5	-36.3 **	345.8	376.1	-30.3*	351.6	375.7	-24.1	348.6	377.2	-28.6*
		(9.4)	(5.9)	(11.0)	(11.1)	(5.6)	(12.4)	(10.2)	(5.7)	(11.7)	(9.7)	(5.8)	(11.3)
**Potassium (mg)**	mean (SE)	237.9	165.5	72.4 ***	204.8	177.9	26.9**	224	172.4	51.5***	236.1	167.7	68.4***
		(7.2)	(4.2)	(8.3)	(7.4)	(4.2)	(8.5)	(7.5)	(4.2)	(8.6)	(7.5)	(4.2)	(8.6)
**Magnesium (mg)**	mean (SE)	59.4	27.2	32.2 ***	51.3	31.4	19.9***	55.4	29.8	25.6***	59	28.1	30.9***
		(2.0)	(0.61)	(2.1)	(1.9)	(0.8)	(1.9)	(2.1)	(0.7)	(2.1)	(2.0)	(0.7)	(2.1)
**Folic acid (mcg)**	mean (SE)	77.1	66.9	10.2	27.3	77.4	-50.1***	42.7	75.5	-32.8**	68.7	69.4	-0.70
		(9.6)	(4.0)	(10.4)	(5.3)	(4.4)	(6.9)	(7.2)	(4.3)	(8.5)	(9.9)	(4.0)	(10.6)
**Thiamine (mg)**	mean (SE)	0.33	0.32	0.01	0.21	0.34	-0.14***	0.25	0.34	-0.09 ***	0.31	0.33	-0.02
		(0.02)	(0.01)	(0.03)	(0.02)	(0.01)	(0.02)	(0.02)	(0.01)	(0.02)	(0.02)	(0.01)	(0.03)
**Riboflavin (mg)**	mean (SE)	0.30	0.29	0.01	0.15	0.32	-0.17***	0.19	0.32	-0.12***	0.27	0.30	-0.04
		(0.03)	(0.01)	(0.03)	(0.01)	(0.01)	(0.02)	(0.02)	(0.01)	(0.02)	(0.03)	(0.01)	(0.03)
**Niacin (mg)**	mean (SE)	4.14	3.60	0.54	2.57	3.95	-1.37***	3.04	3.88	-0.85**	3.83	3.69	0.13
		(0.31)	(0.12)	(0.33)	(0.15)	(0.14)	(0.21)	(0.19)	(0.14)	(0.23)	(0.31)	(0.12)	(0.34)
**Vitamin B6 (mcg)**^d^	mean (SE)	0.40	0.25	0.15***	0.19	0.31	-0.11**	0.28	0.29	-0.01	0.37	0.27	0.10**
		(0.04)	(0.01)	(0.05)	(0.02)	(0.02)	(0.03)	(0.04)	(0.02)	(0.04)	(0.05)	(0.01)	(0.05)
**Vitamin B12 (mcg)**^d^	mean (SE)	0.85	0.55	0.30**	0.29	0.68	-0.39***	0.50	0.64	-0.15	0.73	0.58	0.15
		(0.12)	(0.04)	(0.12)	(0.06)	(0.05)	(0.08)	(0.09)	(0.05)	(0.10)	(0.12)	(0.04)	(0.13)
**Vitamin E (mcg)**	mean (SE)	1.66	1.26	0.40*	1.06	1.41	-0.35	1.19	1.39	-0.20	1.71	1.25	0.46**
		(0.23)	(0.06)	(0.24)	(0.08)	(0.08)	(0.12)	(0.16)	(0.08)	(0.18)	(0.24)	(0.06)	(0.25)
**Zinc (mg)**	mean (SE)	2.35	1.33	1.02***	1.53	1.58	-0.05	1.78	1.52	0.26	2.30	1.37	0.92***
		(0.22)	(0.07)	(0.23)	(0.10)	(0.09)	(0.13)	(0.13)	(0.09)	(0.15)	(0.23)	(0.07)	(0.24)
**Iron (mg)**	mean (SE)	4.66	3.37	1.29***	2.63	3.87	-1.24**	3.26	3.76	-0.51	4.28	3.50	0.77*
		(0.38)	(0.15)	(0.40)	(0.26)	(0.16)	(0.31)	(0.29)	(0.16)	(0.33)	(0.38)	(0.15)	(0.41)

^a^ We defined and applied four different carb ratios based on, per 10g of carb:(a) ≥1g fiber (10:1 carb:fiber), (b) ≥1g fiber and <1g free sugars (10:1:1 carb:fiber:free sugars), (c) ≥1g fiber and <2g free sugars (10:1:2 carb:fiber:free sugars); and (d) ≥1g fiber and, per each 1 g of fiber, <2g free sugars (10:1 carb:fiber, 1:2 fiber:free sugars; or 10:1|1:2).

^b^ The amount of nutrients contained in each of the food code per 100 grams available in NHANES was calculated using US Department of Agriculture’s Food and Nutrient Database for Dietary Studies (FNDDS), corresponding to the 2013–2016 cycles of the National Health and Nutrition Examination Survey (NHANES). All units were expressed per 100 g of food product. Among vitamins and minerals, vitamin B6, vitamin E, zinc and iron were each significantly higher in products that met the10:1 and 10:1|1:2 ratios, compared to products that did not meet these metrics, while thiamine, riboflavin and niacin were each significantly lower in products that met the 10:1:1 and 10:1:2 metrics, compared to products that did not meet these metrics.

^C^ Values represent number (#) and percent (%) of products meeting or not meeting each of the ratios, as well as mean and standard error (SE) of nutrient composition.

^d^ Statistical significance for the difference (Dif.) is noted as ***P<0.001, **P<0.01 and *P<0.05.

^e^ Total sugars included both added sugars and natural sugars such as lactose present in milk and fructose present in whole or cut fruit and 100% fruit juice; added sugars included sugars that were added to foods as an ingredient during preparation, processing, or at the table; and free sugars included added sugars (e.g., honey, white sugar, syrups), sugar present in beverages (excluding natural sugars from dairy), sugars from fruit juice, and sugars from extruded fruit/vegetable products.

In sensitivity analyses, different food categories were separately evaluated. Excluding cold cereals, the different levels of folic acid, thiamine, riboflavin, and niacin among products meeting vs. not meeting the 10:1:2 and 10:1:1 ratios were smaller and were also more similar to findings for the 10:1 and 10:1|1:2 ratios (S3 Table in S1 File). Excluding mixed dishes, 10:1 and 10:1|1:2 ratios performed similarly for identifying products with higher nutritional quality except not as well for folic acid, thiamine, riboflavin, and niacin (S4 Table in S1 File). Findings for each of the individual food categories were shown in (S5–S14 Tables in S1 File); because some of these categories had relatively small numbers of products reported consumed, these subgroup findings should be interpreted in that light.

### Relationships of each metric with selected nutrient profiling systems

**Fig 1:**
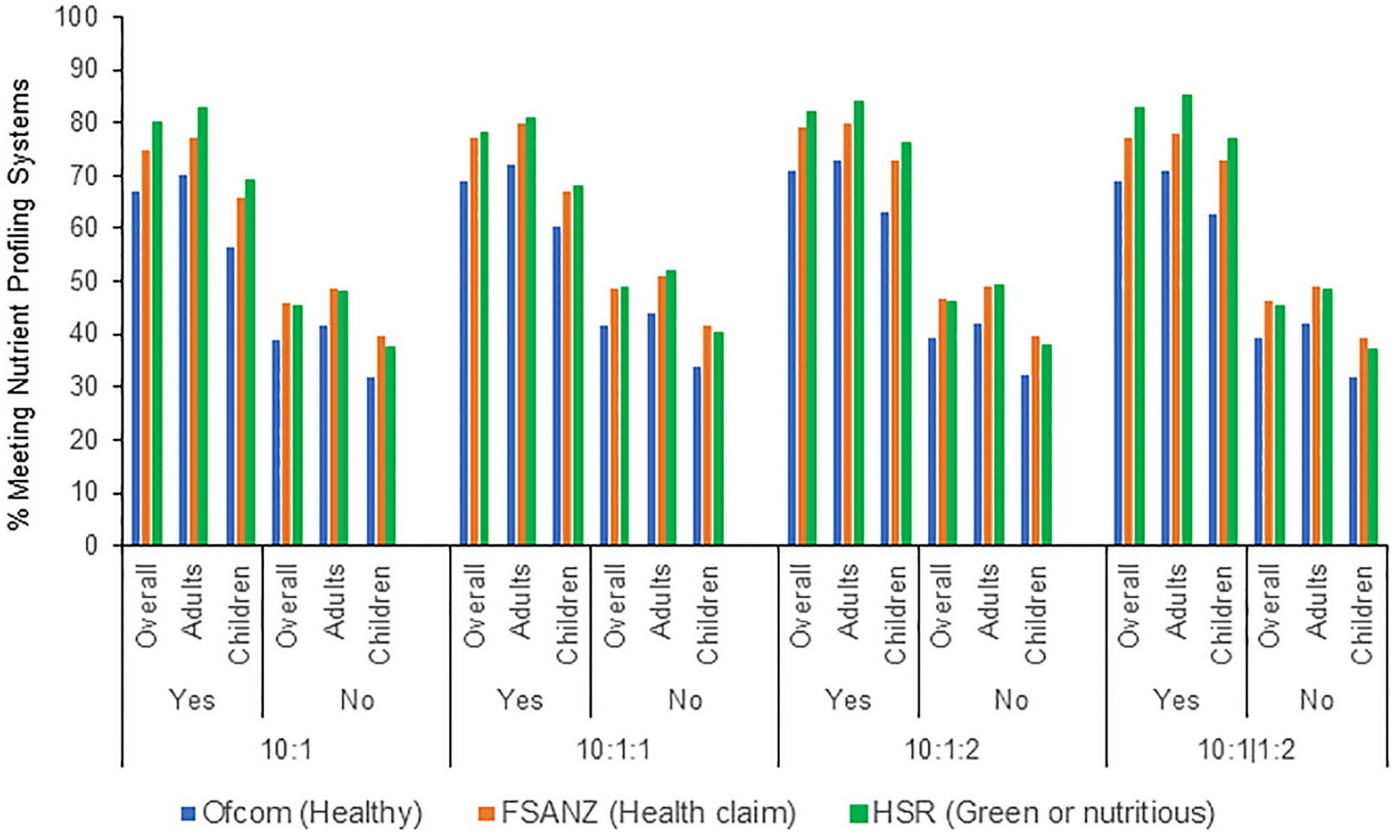
Comparisons of consumed carbohydrate-rich products meeting each of the three nutrient profiling systems classified as either meeting or not meeting each of the four proposed metrics. These four different carb metrics were based on, per 10g of carb:(a) ≥1g fiber (10:1 carb:fiber), (b) ≥1g fiber and <1g free sugars (10:1:1 carb:fiber:free sugars), (c) ≥1g fiber and <2g free sugars (10:1:2 carb:fiber:free sugars); and (d) ≥1g fiber and, per each 1 g of fiber, <2g free sugars (10:1 carb:fiber, 1:2 fiber:free sugars; or 10:1|1:2). Children included individuals aged 2–19 years old and adults included individuals aged 20 years and older. Ofcom model was developed for the regulation of television advertising to children in the United Kingdom. Foods with a final score of <4 points and beverages scoring <1 point are considered as healthy. The FSANZ (Food Standards Australia New Zealand) model was developed for the regulation of health claims on foods in Australia and New Zealand. Foods with a final score of <4 points and beverages scoring <1 point are meeting score criteria to carry a health claim. The HSR (Health Star Rating) is a government led initiative that scores the nutritional value of packaged foods. It is designed to help consumers make healthier choices when shopping within a category. The score of HSR ranges from ½ star to 5 starts. Foods and beverages with a final score of 3.5 points or more is considered as green or nutritious.

When we evaluated how the metrics identified carb-rich products meeting NPS-defined criteria for healthfulness, each of the four metrics generally performed similarly (Fig 1, S2 Table in S1 File). Across the NPSs, about 70–85% of products that met each metric also met desirable thresholds for the NPSs (highest for Health Star Rating, lowest for Ofcom). In contrast, among products that did not meet each metric, only about 38–45% met desirable thresholds for the NPSs. Generally, products consumed by children were less likely to meet the NPSs compared to products consumed by adults. The relationship between each metric and meeting each of the three NPSs across the 12 food categories was shown in S15 Table in S1 File and comparing available carb-rich products meeting each of the three NPSs, each of the four metrics was shown in S16 Table in S1 File.

## Discussion

In this study based on a nationally representative U.S. sample, we investigated how four pragmatic metrics assessed the nutritional quality of 2,208 carb-rich foods and beverages, including the previously validated 10:1 carb:fiber ratio and three additional metrics that further restricted free sugars. Across the four metrics, between 1 in 4 (23.2%; 10:1) and 1 in 6 (16.4%; 10:1:1) products met the criteria. All proposed metrics identified products with generally higher nutritional quality; overall, the 10:1 and 10:1|1:2 ratios appeared to perform best in terms of nutrient differences as well as greater consumer product choices.

Meaningful differences were also identified by food categories. Generally, cold cereals, cooked cereals, cooked grains, breads, and crackers had the largest proportions of products meeting these metrics, while sweet bakery products, quick breads/bread products, and snack/meal bars had the lowest. These findings identify food categories where carbohydrate quality is poor and thus indicate where industry could improve carbohydrate quality of products by increasing the fiber and reducing sugar contents. Although baby food was included in our categorization, our expectation was that few baby foods would meet any of the metrics because formulations may be limited by regulatory guidance [33]. Differences between metrics were also most pronounced in certain food categories. For example, compared to the 10:1 ratio, restricting sugars as a percentage of total carbs (10:1:1, 10:1:2) greatly diminished the number of cold cereals meeting the criteria, while restricting sugars as a ratio to fiber (10:1|1:2) had smaller effects. Restricting the content of free sugars (as opposed to the sum of starch + sugar) also eliminated all smoothies and (except for 10:1|1:2) all snack/meal bars.

The interpretation of the value of these different identifications partly depends on the scientific assessment of relative harms of refined starch vs. sugar. If refined starches vs. sugars in foods are considered metabolically similar for health (e.g., based on glycemic responses and associations with long-term weight gain), then the additional product eliminations may not be useful [34, 35]. If free sugars are considered worse for health than refined starch (which appears true for sugar-sweetened beverages, but more uncertain and controversial for sugars vs. starch in foods), then the 10:1:1, 10:1:2, and 10:1|1:2 ratios may be preferable [7]. Among these latter three, our findings highlight a key question for future research, that is whether biologic effects of sugars should be considered relative to overall carbohydrate (10:1:1, 10:1:2) or to dietary fiber (10:1|1:2).

The 10:1:2 and 10:1:1 ratios identified products with smaller differences in several nutrients (protein, fiber, potassium, magnesium), and lower levels of other nutrients (folic acid, thiamine, riboflavin, niacin), largely due to restriction of cold breakfast cereals. Among such cereals, many that are rich in whole grains also contain some added sugars to increase palatability, while those with no added sugars are often essentially 100% refined starch (e.g. corn starch). These findings raise the important question of focusing on added sugars alone, which may drive consumers and industry formulation toward cereals high in refined starches and low in whole grains. Our results also suggest a need to understand whether consumers will use such labels or any other alternatives to select carb-rich foods overall or within specific subcategories.

To our knowledge, this is the first investigation to evaluate the healthfulness of various carb-rich products as consumed by American adults and children. By all of the metrics, children consumed a smaller proportion of healthier carb-rich products than did adults. These findings provide necessary quantitative data on the conventionally recognized gaps in the nutritional quality of carb-rich products promoted to and consumed by children in the US, including which food categories are in particular need of improvement.

Each of the four metrics performed reasonably well, and choices of which to use may partly depend on which nutrients are prioritized for any particular population or health priority. In addition, most consumers may be unlikely to calculate these metrics for themselves–other than the 10:1 ratio which is most straightforward and some consumers could utilize by seeking at least 1 g of fiber for every 10 g of carbohydrate–so these metrics may be most effective if added to nutrition facts panels or front-of-package labeling by companies or government policies. We did not evaluate the direct associations of these metrics with other factors that might influence nutritional quality, such as the extent of processing that could increase glycemic index or reduce contents of trace phytonutrients, as these factors are not listed on food labels nor commonly available in established nutrient databases. However, based on the relationships of the tested metrics with nutrients such as protein, total sugars, free sugars, added sugars, fiber, sodium, potassium, magnesium, folate, thiamine, riboflavin, niacin, vitamin B6, vitamin B12, vitamin E, zinc and iron, it is reasonable to hypothesize that factors such as hyper-processing (which strips away many of these nutrients), glycemic index (which correlates positively with sugar contents and inversely with fiber), and phytonutrient contents (which are likely to correlate positively with presence of other naturally occurring nutrients in bran and germ like magnesium, thiamine, riboflavin, niacin, vitamin B6, vitamin B12, vitamin E, zinc and iron) are at least partially captured by these metrics. Our findings support the need for future research examining how these metrics relate to these additional nutritional factors in datasets having direct and reliable measures of these factors, as well as the separate and joint relationship of these metrics with other potential dimensions or indices of healthfulness of foods.

In this regard, all the metrics performed well to predict higher proportions of healthier products based on three established NPS currently being utilized by governments to determine health claims or front-of-pack labeling. The similar performance of the four metrics in relation to these NPS likely relates to the relatively limited set of factors assessed by these and most other NPS, which often focus on a smaller number of perceived harmful nutrients (e.g. saturated fat, salt, added sugars) as well as other factors with little evidence for direct health effects (e.g. total fat), with far less focus on beneficial nutrients. Interestingly, for each of the metrics, a smaller proportion of products consumed by U.S. children vs. adults that met each metric also met the NPS criterion. This indicates that children tend to consume more products not meeting the NPS criterion than adults.

Because carbohydrate quality is more relevant for population health than total quantity [34, 36], simple criteria are needed for consumers to identify healthier carb-rich products. No simple method currently exists: all proposed methods require varying use of the Nutrition Facts label and/or the ingredients list [37]. Industry-sponsored logos such as the “whole grain stamp” help identify whole-grain products but these products may also have higher calories and sugar [38], while industry marketing claims like “made with whole grains” or “multi-grain” can be misleading. Each of the ratios assessed in our analysis require the use of the Nutrition Facts label as well as mathematical manipulation. Optimally, such ratios should be translated for the consumer as front-of-pack logos and/or directly listed on the Nutrition Facts. Such ratios may also be useful for other nutrient indices of foods, such as fat quality (e.g. the ratio of unsaturated to saturated fat) or mineral quality (e.g. the ratio of sodium to potassium) and should be investigated in future research.

Our study has several strengths. We evaluated consumed products, overall and weighted by actual consumption, in a large nationally representative sample of adults and children, increasing generalizability. We investigated four pragmatic metrics of carbohydrate quality, including one validated against major grocery products available in the U.S. and U.K. and three adaptations utilizing different approaches to further restrict free sugars. Nutritional quality of products was evaluated and compared across a wide range of indices, including potentially adverse nutrients as well as multiple beneficial minerals and vitamins that may be consumed in grain products; as well as three NPS currently used by governments.

Potential limitations should be considered. To enable cross-category comparisons, all foods were standardized to 100 g, and actual amounts consumed may vary by product or individual consumer. Nonetheless, our standardized assessment provides the most objective comparison across different products and people. Whole grain foods may contain hundreds or thousands of other trace phytochemicals, yet these could not be evaluated due to absence of widely accepted health effects or relevant standardized nutrient databases. The different metrics were validated against nutritional contents and selected major NPSs (although not others such as SAIN-LIM–score of nutritional adequacy of individual foods -limited nutrient score or SENS—simplified nutrient profiling system), and our finding support the need for future studies of validation against clinical disease endpoints. However, studies of clinical outcomes will be challenging, as studies utilizing 24-hour recalls will accurately capture groups means and averages (as in the present study) but not any individual person’s habitual dietary consumption (essential for correlation to their clinical events); while studies utilizing food frequency questionnaires assess habitual consumption but lack the granularity of product information to accurately define these metrics for any person. In most countries, package labels do not distinguish type (soluble insoluble) or source (naturally occurring, added) of fiber. While evidence for health benefits of higher total dietary fiber intake is largely consistent [34], the health benefits of isolated or synthetic fibers vary considerably depending on the physiological properties of the fiber [39]. Under new FDA regulations [40], dietary fiber on the Nutrition Facts label must have a physiological health benefit although it does not specify added versus naturally occurring fiber. We assessed free sugars, and results could slightly differ if based on the (smaller) subset of added sugars. The evaluated metrics did not aim to assess every aspect of food quality, but to identify a simple, pragmatic, and useful metric of carbohydrate quality. For example, we did not assess the relation of these metrics with food processing, glycemic load, phenolic contents, or AGE contents, as these are not available on the Nutrient Facts label for consumers to evaluate, nor are available in most nutrient databases for research investigation. Our findings support the need for future studies to assess whether adding such novel measures to the 10:1 or other ratios evaluated here improves their discrimination of nutritional quality.

## Conclusions

Simple, pragmatic metrics utilizing ratios of carbohydrate, fiber, and in some cases sugars can identify carb-rich products with better nutritional quality. Among these, the 10:1 carb:fiber ratio and the 10:1|1:2 carb:fiber| fiber:free sugars ratio appeared to capture a better overall nutritional profile, although these metrics did differ slightly with respect to nutritional quality within food categories, and capture the largest numbers of consumer choices. These novel findings inform dietary guidance for consumers and the development of policy strategies and industry reformulations to create healthier carb-rich foods.

## Supporting information

S1 Checklist STROBE statement—Checklist of items that should be included in reports of observational studies. (DOCX)

S1 Fig Food items flow chart. (DOCX)

S1 File (DOCX)
