# Supplementary material for: A comparison of different practical indices for assessing carbohydrate quality among carbohydrate-rich processed products in the US
Source: PLoS One. 2020 May 21;15(5):e0231572. doi: 10.1371/journal.pone.0231572 (PMC7241725; doi:10.1371/journal.pone.0231572)
Supplement: S1 Fig — (DOCX) [file pone.0231572.s002.docx]

Supplemental Figure 1. Food items flow chart

Food codes

FNDDS 2013-2014

FNDDS 2015-2016

FNDDS version by NHANES survey cycle

N=8,536

N=8,690

N=9,514

Combined total food codes

N=2,208

Included food codes, i.e. carbohydrate-rich food products for the analysis
