# Supplementary material for: A comparison of different practical indices for assessing carbohydrate quality among carbohydrate-rich processed products in the US
Source: PLoS One. 2020 May 21;15(5):e0231572. doi: 10.1371/journal.pone.0231572 (PMC7241725; doi:10.1371/journal.pone.0231572)
Supplement: S1 File — (DOCX) [file pone.0231572.s003.docx]

**Supplemental Material**

This supplemental material has been provided by the authors to give readers additional information about their work.

Supplement to: Junxiu Liu; Colin D Rehm; Peilin Shi; Nicola M McKeown; Dariush Mozaffarian; Renata Micha. A comparison of different metrics and nutrient profiling systems for assessing carbohydrate quality in the US

**Contents**

[eText Nutrient profiling models 3](#_Toc13840245)

[*UK FSA/Ofcom model* 3](#_Toc13840246)

[*FSANZ model* 3](#_Toc13840247)

[*Healthy Star Rating model* 4](#_Toc13840248)

[Table S1. Correlation matrix among four proposed metrics for evaluation of available carbohydrate-rich products meeting each of four proposed metrics for assessing carbohydrate quality 5](#_Toc13840249)

[Table S2. Comparisons of consumed carbohydrate-rich products meeting each of three nutrient profiling systems by meeting or not meeting each four proposed metrics 6](#_Toc13840250)

[Table S3. Nutrient composition of available carbohydrate-rich products -excluding ready to eat cereals- meeting or not meeting each of four proposed metrics for assessing carbohydrate quality in the US 8](#_Toc13840251)

[Table S4. Nutrient composition of available carbohydrate-rich products -excluding mixed dishes- meeting or not meeting each of four proposed metrics for assessing carbohydrate quality in the US 10](#_Toc13840252)

[Table S5. Nutrient composition of available baby food-cereals, snacks and sweets meeting or not meeting each of four proposed metrics for assessing carbohydrate quality in the US 12](#_Toc13840253)

[Table S6. Nutrient composition of available bread, rolls, and tortillas meeting or not meeting each of four proposed metrics for assessing carbohydrate quality in the US. 14](#_Toc13840254)

[Table S7. Nutrient composition of available cooked cereals meeting or not meeting each of four proposed metrics for assessing carbohydrate quality in the US. 16](#_Toc13840255)

[Table S8. Nutrient composition of available cooked grains products meeting or not meeting each of four proposed metrics for assessing carbohydrate quality in the US. 18](#_Toc13840256)

[Table S9. Nutrient composition of available crackers products meeting or not meeting each of four proposed metrics for assessing carbohydrate quality in the US 20](#_Toc13840257)

[Table S10. Nutrient composition of available quick breads and bread products meeting or not meeting each of four proposed metrics for assessing carbohydrate quality in the US. 22](#_Toc13840258)

[Table S11. Nutrient composition of available cold cereals products meeting or not meeting each of four proposed metrics for assessing carbohydrate quality in the US 24](#_Toc13840259)

[Table S12. Nutrient composition of available savory snacks products meeting or not meeting each of four proposed metrics for assessing carbohydrate quality in the US 26](#_Toc13840260)

[Table S13. Nutrient composition of available sweet bakery products meeting or not meeting each of four proposed metrics for assessing carbohydrate quality in the US 28](#_Toc13840261)

[Table S14. Nutrient composition of mixed dishes products meeting or not meeting each of four proposed metrics for assessing carbohydrate quality in the US 30](#_Toc13840262)

[Table S15. Comparisons of available and consumed carbohydrate-rich projects meeting each of the three nutrient profiling systems in the US 32](#_Toc13840263)

[Table S16. Comparisons of available carbohydrate-rich projects meeting each of three nutrient profiling systems by meeting or not meeting each four proposed metrics 34](#_Toc13840264)

Supplemental Figure 1. Food items flow chart……………………………………………………….39

# **eText Nutrient profiling models**

Nutrient profiling is defined by the World Health Organization (WHO) as “the science of classifying or ranking foods according to their nutritional composition for reasons related to preventing disease and promoting health”. ^1^ Nutrient profile (NP) is operationalized by NP models through algorithms that take into consideration the amounts or the presence of nutrients and other components of foods into classifications or scores for rating or evaluating the nutritional quality of foods. A recent systematic review on existing NP models revealed that various NP models are available.^2^ These models are increasingly used by government bodies worldwide to underpin nutrition related policies. Here in this paper, we have applied three NP models including Ofcom, and summarized as follows.

## ***UK FSA/Ofcom model***

The UK Food Standards Agency (FSA) Ofcom model was developed in 2004-2005 as a tool to differentiate of foods on the basis of their nutritional composition for regulating the marketing of foods to children.^3^ In this model, a single score based on a set of “negative” indicators (energy, saturated fat, sugars and sodium) is counter-balanced by a score based on ‘positive’ indicators (protein, fiber, and ‘fruits, vegetables and nuts’). All measurement criteria were per 100 grams rather than actual serving size. This is justified on the basis that the model is designed to measure the nutritional quality of the food regardless of the way it is eaten.

As said, the model provides a single score for any given food product, which is calculated by the number of points for ‘negative’ nutrients offset by points for ‘positive’ nutrients. Points are allocated on the basis of the nutritional content in 100g of a food or drink. The model consists of two food categories and applies equally to all food and drinks with no exemptions or category-specific criteria. Details for scoring algorithm were provided elsewhere. ^3,4^

Foods scoring 4 or more points, and drinks scoring 1 or more points, are classified as ‘less healthy’ and are subject to Ofcom’s controls on the advertising of foods or drinks to children on television.

## ***FSANZ model***

The Food Standards Australia New Zealand (FSANZ) model applied the Nutrient Profiling Scoring Criterion (NPSC) to determine whether a product is eligible to carry a health claim based on its nutrient profile in Australia and New Zealand. Details of scoring algorithm were available elsewhere.^5,6^ The FSANZ NP models was chosen because it was specifically developed to determine the eligibility of a product to carry health claims and is already enforced in Australia and New Zealand. FSANZ divides foods into three categories including beverages (category 1); cheese, oil, margarine and butter (category 3); and any food other than those included in the previous two categories (category 2). The baseline points for energy (0-10 points), saturated fat (0-30 points), total sugars (0-10 points), sodium (0-30 points) is then calculated for each product. Then V points based on content of fruits, vegetables, nuts and legumes, P points based on protein, and F points based on fiber were calculated. The final NPSC score was calculated using the formula as: Final score = Baseline points – (V points) – (P points) – (F points). To carry a health claim, the NPSC score must be less than 1 for category 1, less than 4 for category 2 and less than 28 for category 3. Lower scores indicate a healthier product.

## ***Healthy Star Rating model***

Derived from the FSANZ NPSC system, Australia and New Zealand adopted a voluntary front-of-pack nutrition labeling scheme in the form of the Health Star Rating (HSR) system in 2014. The HSR scoring algorithm was developed by FSANZ in consultation with technical and nutrition experts, including industry representation.^7^ The HSR system is designed to assist consumers to discriminate between foods in the same food category and to compare across different food categories. The details of scoring algorithm were documented elsewhere. ^8^ In brief, foods contain six categories (i.e., non-dairy beverages; dairy beverages; oils and spreads; cheese and processed cheese; all other dairy foods; and all other non-dairy foods). Ratings are determined by an overall assessment of ‘negative’ components (total energy, total sugars, saturated fat and sodium) and ‘positive’ components of foods (protein, fiber, and fruit, vegetable, nut and legume content (FVNL)). Baseline points were calculated based on the energy, saturated fat, total sugar and sodium contents per 100 g. Then modifying pointes were calculated for FVNL, protein and fiber where applicable. A HSR score was summarized by subtracting the modifying points from baseline points. The HSR algorithm generates 10 different star rating from 0.5 (least healthy) to 5.0 stars (most healthy) in half-star increment. with a possibility of 10 different star ratings. A higher HSR reflects a healthier product.

# **Table S1. Correlation matrix among four proposed metrics for evaluation of available carbohydrate-rich products meeting each of four proposed metrics for assessing carbohydrate quality.^a^**

| **Four proposed metrics** | **Four proposed metrics** | | | |
| --- | --- | --- | --- | --- |
|  | **10:1** | **10:1:1** | **10:1:2** | **10:1\|1:2** |
| **10:1** | 1 |  |  |  |
| **10:1:1** | 0.79 | 1 |  |  |
| **10:1:2** | 0.88 | 0.91 | 1 |  |
| **10:1\|1:2** | 0.95 | 0.84 | 0.93 | 1 |

^a^ We defined and applied four different carb ratios: per 10g of carb:(a) ≥1g fiber (10:1 carb:fiber), (b) ≥1g fiber and <1g free sugars (10:1:1 carb:fiber:free sugars), (c) ≥1g fiber and <2g free sugars (10:1:2 carb:fiber:free sugars); and (d) ≥1g fiber and, per each 1 g of fiber, <2g free sugars (10:1 carb:fiber, 1:2 fiber:free sugars; or 10:1|1:2).

# **Table S2. Comparisons of consumed carbohydrate-rich products meeting each of three nutrient profiling systems by meeting or not meeting each four proposed metrics**

| **Four metrics^a^** | **Analysis^b^** | **Products** | |  | **% Meeting Nutrient Profiling Systems^d^** | | |
| --- | --- | --- | --- | --- | --- | --- | --- |
|  |  | **#** | **Frequency^c^**  **(million)** |  | **Ofcom**  **(Healthy)** | **FSANZ**  **(Health claim)** | **HSR**  **(Green or nutritious)** |
| **10:1** |  |  |  |  |  |  |  |
| Yes |  |  |  |  |  |  |  |
|  | *Overall* | 383 | 615.6 |  | 67.0 | 75.0 | 80.0 |
|  | *Adults* | 350 | 481.9 |  | 70.0 | 77.0 | 83.0 |
|  | *Children* | 287 | 133.7 |  | 56.4 | 66.0 | 69.0 |
| No |  |  |  |  |  |  |  |
|  | *Overall* | 1353 | 2696.5 |  | 38.9 | 46.1 | 45.2 |
|  | *Adults* | 1271 | 1967.5 |  | 41.5 | 48.5 | 48.0 |
|  | *Children* | 1043 | 729.0 |  | 32.1 | 39.6 | 37.6 |
| **10:1:1** |  |  |  |  |  |  |  |
| Yes |  |  |  |  |  |  |  |
|  | *Overall* | 258 | 320.1 |  | 69.0 | 77.0 | 78.0 |
|  | *Adults* | 233 | 250.3 |  | 72.0 | 80.0 | 81.0 |
|  | *Children* | 184 | 69.8 |  | 60.4 | 67.0 | 68.0 |
| No |  |  |  |  |  |  |  |
|  | *Overall* | 1478 | 2991.9 |  | 41.5 | 48.6 | 48.8 |
|  | *Adults* | 1388 | 2199.1 |  | 44.2 | 51.2 | 51.9 |
|  | *Children* | 1146 | 792.8 |  | 33.7 | 41.6 | 40.3 |
| **10:1:2** |  |  |  |  |  |  |  |
| Yes |  |  |  |  |  |  |  |
|  | *Overall* | 308 | 491.8 |  | 71.0 | 79.0 | 82.0 |
|  | *Adults* | 281 | 388.6 |  | 73.0 | 80.0 | 84.0 |
|  | *Children* | 225 | 103.2 |  | 63.1 | 73.0 | 76.0 |
| No |  |  |  |  |  |  |  |
|  | *Overall* | 1428 | 2820.3 |  | 39.4 | 46.7 | 46.3 |
|  | *Adults* | 1340 | 2060.8 |  | 42.1 | 49.2 | 49.4 |
|  | *Children* | 1105 | 759.4 |  | 32.2 | 39.6 | 38.1 |
| **10:1\|1:2** |  |  |  |  |  |  |  |
| Yes |  |  |  |  |  |  |  |
|  | *Overall* | 348 | 546.7 |  | 69.0 | 77.0 | 83.0 |
|  | *Adults* | 318 | 433.8 |  | 71.0 | 78.0 | 85.0 |
|  | *Children* | 256 | 112.9 |  | 62.7 | 73.0 | 77.0 |
| No |  |  |  |  |  |  |  |
|  | *Overall* | 1388 | 2765.4 |  | 39.2 | 46.3 | 45.4 |
|  | *Adults* | 1303 | 2015.7 |  | 41.9 | 48.9 | 48.4 |
|  | *Children* | 1074 | 749.7 |  | 31.9 | 39.3 | 37.3 |

**^a^**We defined and applied four different carb metrics: per 10g of carb:(a) ≥1g fiber (10:1 carb:fiber), (b) ≥1g fiber and <1g free sugars (10:1:1 carb:fiber:free sugars), (c) ≥1g fiber and <2g free sugars (10:1:2 carb:fiber:free sugars); and (d) ≥1g fiber and, per each 1 g of fiber, <2g free sugars (10:1 carb:fiber, 1:2 fiber:free sugars; or 10:1|1:2).

^b^ Children included individuals aged 2-19 years old and adults included individuals aged 20 years and older.

^c^Frequency means that products were weighted by their actual reported consumption levels overall or by children or adults, with products consumed more frequently receiving greater weight.

^d^ Ofcom model was developed for the regulation of television advertising to children in the United Kingdom. Foods with a final score of <4 points and beverages scoring <1 point are considered as healthy. The FSANZ (Food Standards Australia New Zealand) model was developed for the regulation of health claims on foods in Australia and New Zealand. Foods with a final score of <4 points and beverages scoring <1 point are meeting score criteria to carry a health claim. The HSR (Health Star Rating) is a government led initiative that scores the nutritional value of packaged foods. It is designed to help consumers make healthier choices when shopping within a category. The score of HSR ranges from ½ star to 5 stars. Foods and beverages with a final score of 3.5 points or more is considered as green or nutritious.

# **Table S3. Nutrient composition of available carbohydrate-rich products -excluding ready to eat cereals- meeting or not meeting each of four proposed metrics for assessing carbohydrate quality in the US**

|  |  | **Meeting or not Meeting each Metric**^s^ | | | | | | | | | | | |
| --- | --- | --- | --- | --- | --- | --- | --- | --- | --- | --- | --- | --- | --- |
| **Composition**^b^ | **Weighted**  **Values**^c^ | **10:1** | | | **10:1:1** | | | **10:1:2** | | | **10:1\|1:2** | | |
|  |  | **Yes** | **No** | **Dif.**^d^ | **Yes** | **No** | **Dif.** ^d^ | **Yes** | **No** | **Dif.** ^d^ | **Yes** | **No** | **Dif.** ^d^ |
|  | # (%) products | 425  (21.1%) | 1585  (78.9%) |  | 344  (17.1%) | 1666  (82.9%) |  | 382  (19.0%) | 1628  (81%) |  | 403  (20%) | 1607  (80%) |  |
| **Calories**  **(kJ)** | mean (SE) | 222.2  (6.9) | 265  (3.4) | **-**42.8***  (7.7) | 214.4  (7.7) | 264.6  (3.3) | **-**50.2***  (8.3) | 219.7  (7.2) | 264.5  (3.4) | **-**44.7***  (7.9) | 221.6  (7.0) | 264.6  (3.4) | **-**43***  (7.8) |
| **Total fat**  **(g)** | mean (SE) | 7.67  (0.41) | 9.58  (0.21) | **-**1.91***  (0.46) | 7.71  (0.48) | 9.48  (0.20) | **-**1.77***  (0.52) | 7.69  (0.44) | 9.52  (0.21) | **-**1.83***  (0.49) | 7.71  (0.42) | 9.54  (0.21) | **-**1.84***  (0.47) |
| **Saturated fat (g)** | mean (SE) | 2.15  (0.16) | 2.74  (0.07) | **-**0.60***  (0.18) | 2.18  (0.19) | 2.71  (0.07) | **-**0.53**  (0.20) | 2.13  (0.17) | 2.73  (0.07) | **-**0.61***  (0.18) | 2.12  (0.16) | 2.74  (0.07) | **-**0.63***  (0.18) |
| **Protein**  **(g)** | mean (SE) | 7.02  (0.19) | 6.05  (0.09) | 0.97***  (0.21) | 6.84  (0.19) | 6.13  (0.09) | 0.71**  (0.21) | 7.07  (0.19) | 6.06  (0.09) | 1.0***  (0.21) | 7.13  (0.20) | 6.04  (0.09) | 1.09***  (0.22) |
| **Total sugar (g)**^d^ | mean (SE) | 4.23  (0.33) | 10.41  (0.32) | **-**6.18***  (0.46) | 2.2  (0.16) | 10.53  (0.31) | **-**8.33***  (0.35) | 2.73  (0.18) | 10.6  (0.31) | **-**7.87***  (0.36) | 3.29  (0.24) | 10.56  (0.32) | **-**7.27***  (0.39) |
| **Added sugar (g)**^d^ | mean (SE) | 2.39  (0.30) | 8.11  (0.30) | **-**5.73***  (0.60) | 0.5  (0.06) | 8.22  (0.29) | **-**7.72***  (0.64) | 1.03  (0.10) | 8.28  (0.30) | **-**7.26***  (0.61) | 1.6  (0.19) | 8.23  (0.30) | **-**6.63***  (0.60) |
| **Free sugar (g)**^d^ | mean (SE) | 2.5  (0.30) | 8.2  (0.30) | **-**5.7***  (0.42) | 0.51  (0.06) | 8.33  (0.29) | **-**7.83***  (0.30) | 1.03  (0.10) | 8.4  (0.30) | **-**7.37***  (0.32) | 1.61  (0.19) | 8.35  (0.30) | **-**6.74***  (0.36) |
| **Fiber**  **(g)** | mean (SE) | 5.05  (0.19) | 1.89  (0.03) | 3.16***  (0.20) | 4.83  (0.21) | 2.09  (0.05) | 2.74***  (0.21) | 4.90  (0.19) | 2.01  (0.04) | 2.90***  (0.20) | 5.04  (0.20) | 1.93  (0.04) | 3.11***  (0.20) |
| **Sodium**  **(mg)** | mean (SE) | 343.9  (10.2) | 370.2  (6.1) | **-**26.4*  (11.9) | 349.5  (11.3) | 367.8  (5.9) | 18.2  (12.8) | 357.4  (10.7) | 366.4  (6.0) | 8.9  (12.3) | 353.2  (10.4) | 367.5  (6.0) | 14.3  (12.1) |
| **Potassium (mg)** | mean (SE) | 203.7  (6.6) | 163.6  (4.5) | 40***  (8.0) | 192.5  (7.0) | 167.9  (4.4) | 24.6*  (8.3) | 200.7  (6.8) | 165.4  (4.4) | 35.4***  (8.1) | 202.8  (6.9) | 164.4  (4.4) | 38.4***  (8.2) |
| **Magnesium (mg)** | mean (SE) | 50.3  (1.8) | 25.9  (0.62) | 24.4***  (1.9) | 47.6  (1.8) | 27.6  (0.7) | 20***  (1.9) | 49.1  (1.7) | 26.8  (0.7) | 22.3***  (1.8) | 50.1  (1.7) | 26.3  (0.6) | 23.8***  (1.8) |
| **Folic acid**  **(mcg)** | mean (SE) | 12.4  (1.5) | 32.3  (1.0) | **-**19.9***  (1.8) | 11  (1.2) | 31.6  (1.0) | **-**20.6***  (1.5) | 10.8  (1.1) | 32.2  (1.0) | **-**21.4***  (1.5) | 12.3  (1.6) | 32.1  (0.9) | **-**19.8***  (1.9) |
| **Thiamine**  **(mg)** | mean (SE) | 0.19  (0.01) | 0.25  (0.01) | **-**0.06***  (0.01) | 0.17  (0.01) | 0.25  (0.01) | **-**0.07***  (0.01) | 0.18  (0.01) | 0.25  (0.01) | **-**0.06***  (0.01) | 0.19  (0.01) | 0.24  (0.01) | **-**0.06***  (0.01) |
| **Riboflavin**  **(mg)** | mean (SE) | 0.15  (0.01) | 0.21  (0.01) | **-**0.06*** (0.01) | 0.13  (0.01) | 0.21  (0.01) | **-**0.08***  (0.01) | 0.14  (0.01) | 0.21  (0.01) | **-**0.07***  (0.01) | 0.15  (0.01) | 0.21  (0.01) | **-**0.07***  (0.01) |
| **Niacin**  **(mg)** | mean (SE) | 2.28  (0.1) | 2.6  (0.07) | **-**0.32*  (0.12) | 2.15  (0.09) | 2.61  (0.07) | **-**0.46*  (0.11) | 2.24  (0.09) | 2.6  (0.07) | **-**0.36*  (0.11) | 2.32  (0.1) | 2.58  (0.07) | **-**0.26  (0.12) |
| **Vitamin B6 (mcg)**^d^ | mean (SE) | 0.15  (0.01) | 0.13  (0.01) | -0.02  (0.01) | 0.13  (0.001) | 0.14  (0.01) | **-**0.01  (0.01) | 0.14  (0.001) | 0.14  (0.01) | 0  (0.01) | 0.15  (0.01) | 0.13  (0.01) | 0.02  (0.01) |
| **Vitamin B12 (mcg)**^d^ | mean (SE) | 0.17  (0.02) | 0.21  (0.01) | **-**0.04  (0.03) | 0.13  (0.01) | 0.22  (0.01) | **-**0.09**  (0.02) | 0.15  (0.02) | 0.22  (0.01) | **-**0.06*  (0.03) | 0.17  (0.02) | 0.21  (0.01) | **-**0.05  (0.03) |
| **Vitamin E**  **(mcg)** | mean (SE) | 1.07  (0.07) | 1.14  (0.04) | **-**0.07  (0.09) | 1.03  (0.07) | 1.15  (0.04) | **-**0.12  (0.1) | 1.03  (0.07) | 1.15  (0.05) | **-**0.12  (0.08) | 1.09  (0.08) | 1.14  (0.04) | **-**0.05  (0.09) |
| **Zinc**  **(mg)** | mean (SE) | 1.26  (0.04) | 0.82  (0.03) | 0.44***  (0.05) | 1.24  (0.04) | 0.85  (0.02) | 0.40***  (0.05) | 1.27  (0.04) | 0.83  (0.02) | 0.44***  (0.05) | 1.28  (0.04) | 0.82  (0.02) | 0.46***  (0.05) |
| **Iron**  **(mg)** | mean (SE) | 2.05  (0.13) | 2.33  (0.1) | **-**0.28  (0.16) | 1.92  (0.15) | 2.35  (0.09) | **-**0.42  (0.18) | 2.0  (0.14) | 2.34  (0.1) | **-**0.34  (0.17) | 2.06  (0.13) | 2.33  (0.10) | **-**0.26  (0.16) |

^a^ We defined and applied four different carb ratios: per 10g of carb:(a) ≥1g fiber (10:1 carb:fiber), (b) ≥1g fiber and <1g free sugars (10:1:1 carb:fiber:free sugars), (c) ≥1g fiber and <2g free sugars (10:1:2 carb:fiber:free sugars); and (d) ≥1g fiber and, per each 1 g of fiber, <2g free sugars (10:1 carb:fiber, 1:2 fiber:free sugars; or 10:1|1:2).

^b^ All units were expressed as per 100 grams.

^c^ The amount of nutrients contained in each of the food code per 100 grams available in NHANES was calculated using US Department of Agriculture’s Food and Nutrient Database for Dietary Studies.

^d^ Statistical significances for differences (Dif.) is noted as ****P*<0.001, ***P*<0.01 and **P*<0.05.

^e^ Total sugars included both added sugar and natural sugar such as lactose present in milk and fructose present in whole or cut fruit and 100% fruit juice; added sugars included sugars that were added to foods as an ingredient during preparation, processing, or at the table; and free sugars included added sugars (e.g., honey, white sugar, syrups), sugar present in beverages (excluding sugars from dairy), sugar from fruit juice, and sugars from extruded fruit/vegetable products.

# **Table S4. Nutrient composition of available carbohydrate-rich products -excluding mixed dishes- meeting or not meeting each of four proposed metrics for assessing carbohydrate quality in the US**

|  |  | **Meeting or not Meeting each Metric**^s^ | | | | | | | | | | | |
| --- | --- | --- | --- | --- | --- | --- | --- | --- | --- | --- | --- | --- | --- |
| **Composition**^b^ | **Weighted**  **Values**^c^ | **10:1** | | | **10:1:1** | | | **10:1:2** | | | **10:1\|1:2** | | |
|  |  | **Yes** | **No** | **Dif.**^d^ | **Yes** | **No** | **Dif.** ^d^ | **Yes** | **No** | **Dif.** ^d^ | **Yes** | **No** | **Dif.** ^d^ |
|  | # (%) products |  |  |  |  |  |  |  |  |  |  |  |  |
| **Calories**  **(kJ)** | mean (SE) | 280  (7.7) | 315.3  (3.7) | **-**35.3***  (8.5) | 262.7  (10.7) | 314.5  (3.4) | **-**51.8***  (11.3) | 272.2  (9.1) | 314.6  (3.5) | **-**42.5***  (9.8) | 277.5  (8.2) | 314.8  (3.6) | **-**37.3***  (9.0) |
| **Total fat**  **(g)** | mean (SE) | 7.5  (0.47) | 10.5  (0.25) | **-**3.0***  (0.53) | 8.25  (0.70) | 10.06  (0.23) | **-**1.81**  (0.73) | 7.88  (0.58) | 10.21  (0.24) | **-**2.33***  (0.63) | 7.64  (0.51) | 10.36  (0.25) | **-**2.73***  (0.57) |
| **Saturated fat (g)** | mean (SE) | 1.89  (0.18) | 2.92  (0.09) | **-**1.03***  (0.20) | 2.11  (0.27) | 2.78  (0.08) | **-**0.67**  (0.28) | 1.93  (0.22) | 2.85  (0.09) | **-**0.92***  (0.23) | 1.83  (0.19) | 2.91  (0.09) | **-**1.07***  (0.21) |
| **Protein**  **(g)** | mean (SE) | 7.76  (0.23) | 5.98  (0.09) | 1.79***  (0.25) | 7.42  (0.26) | 6.22  (0.10) | 1.2***  (0.28) | 7.77  (0.24) | 6.09  (0.10) | 1.68***  (0.26) | 7.91  (0.24) | 6  (0.09) | 1.92***  (0.26) |
| **Total sugar (g)**^d^ | mean (SE) | 8.53  (0.54) | 15.31  (0.41) | **-**6.78*** (0.68) | 2.66  (0.28) | 15.58  (0.38) | **-**12.92***  (0.47) | 4.58  (0.38) | 15.71  (0.39) | **-**11.13***  (0.54) | 6.52  (0.45) | 15.61  (0.40) | **-**9.09***  (0.61) |
| **Added sugar (g)**^d^ | mean (SE) | 6.07  (0.49) | 12.7  (0.4) | **-**6.63***  (0.77) | 0.68  (0.09) | 12.91  (0.37) | **-**12.23***  (0.9) | 2.27  (0.23) | 13.08  (0.38) | **-**10.82***  (0.82) | 4.18  (0.36) | 12.98  (0.39) | **-**8.8***  (0.79) |
| **Free sugar (g)**^d^ | mean (SE) | 6.2  (0.49) | 12.81  (0.40) | **-**6.61***  (0.63) | 0.68  (0.09) | 13.04  (0.37) | **-**12.36***  (0.38) | 2.27  (0.23) | 13.22  (0.38) | **-**10.95***  (0.45) | 4.17  (0.36) | 13.12  (0.39) | **-**8.95***  (0.53) |
| **Fiber**  **(g)** | mean (SE) | 7.75  (0.29) | 2.36  (0.05) | 5.39***  (0.18) | 6.83  (0.34) | 3.07  (0.09) | 3.75***  (0.35) | 7.16  (0.30) | 2.84  (0.08) | 4.32***  (0.31) | 7.82  (0.31) | 2.52  (0.06) | 5.31***  (0.32) |
| **Sodium**  **(mg)** | mean (SE) | 339.8  (12) | 387.6  (7.5) | **-**47.8**  (14.1) | 345.2  (15.8) | 381.7  (7.0) | **-**36.5*  (17.2) | 351.7  (13.9) | 381.9  (7.2) | **-**30.2  (15.6) | 347.1  (12.8) | 384.2  (7.3) | **-**37.1*  (14.7) |
| **Potassium (mg)** | mean (SE) | 257.4  (9.5) | 178.8  (5.7) | 78.6***  (11.1) | 218.4  (11.4) | 193.5  (5.5) | 24.9  (12.6) | 243  (10.8) | 187.1  (5.6) | 55.8***  (12.1) | 257.3  (10.3) | 181.4  (5.6) | 75.9***  (11.7) |
| **Magnesium (mg)** | mean (SE) | 70.4  (2.6) | 30.5  (0.8) | 39.9***  (2.7) | 64  (2.7) | 35.7  (1.0) | 28.3***  (2.9) | 67.8  (2.7) | 33.7  (0.9) | 34.1***  (2.9) | 71.1  (2.6) | 31.6  (0.9) | 39.5***  (2.8) |
| **Folic acid**  **(mcg)** | mean (SE) | 103.6  (13) | 83.2  (5.4) | 20.4  (14.1) | 38.8  (8.4) | 96.2  (5.8) | -57.4***  (10.2) | 59.8  (10.7) | 94  (5.8) | **-**34.2*  (12.2) | 94.9  (13.9) | 86.2  (5.4) | -8.7  (14.9) |
| **Thiamine**  **(mg)** | mean (SE) | 0.41  (0.03) | 0.37  (0.01) | 0.04  (0.04) | 0.25  (0.02) | 0.40  (0.01) | -0.15***  (0.03) | 0.31  (0.02) | 0.4  (0.01) | **-**0.09**  (0.03) | 0.38  (0.03) | 0.38  (0.01) | 0  (0.04) |
| **Riboflavin**  **(mg)** | mean (SE) | 0.37  (0.04) | 0.36  (0.01) | 0.01  (0.04) | 0.17  (0.02) | 0.39  (0.02) | -0.22***  (0.02) | 0.23  (0.02) | 0.39  (0.02) | **-**0.16***  (0.03) | 0.33  (0.04) | 0.37  (0.01) | **-**0.04  (0.04) |
| **Niacin**  **(mg)** | mean (SE) | 4.85  (0.42) | 4.19  (0.16) | 0.66  (0.45) | 2.79  (0.24) | 4.6  (0.18) | -1.81***  (0.30) | 3.44  (0.27) | 4.54  (0.18) | **-**1.1**  (0.33) | 4.5  (0.44) | 4.3  (0.16) | 0.20  (0.47) |
| **Vitamin B6 (mcg)**^d^ | mean (SE) | 0.51  (0.06) | 0.31  (0.02) | 0.20***  (0.06) | 0.24  (0.03) | 0.38  (0.02) | **-**0.14*  (0.04) | 0.36  (0.06) | 0.36  (0.02) | 0  (0.06) | 0.48  (0.06) | 0.33  (0.02) | 0.15**  (0.07) |
| **Vitamin B12 (mcg)**^d^ | mean (SE) | 1.07  (0.16) | 0.66  (0.05) | 0.41**  (0.17) | 0.32  (0.09) | 0.82  (0.06) | **-**0.51**  (0.11) | 0.61  (0.13) | 0.78  (0.06) | **-**0.18  (0.14) | 0.94  (0.17) | 0.7  (0.05) | 0.23  (0.18) |
| **Vitamin E**  **(mcg)** | mean (SE) | 1.95  (0.31) | 1.5  (0.09) | 0.44  (0.33) | 1.16  (0.12) | 1.68  (0.11) | **-**0.53**  (0.16) | 1.35  (0.23) | 1.66  (0.11) | **-**0.32  (0.26) | 2.06  (0.35) | 1.49  (0.09) | 0.57*  (0.36) |
| **Zinc**  **(mg)** | mean (SE) | 2.86  (0.3) | 1.54  (0.09) | 1.32***  (0.31) | 1.84  (0.16) | 1.85  (0.11) | **-**0.01  (0.20) | 2.15  (0.19) | 1.78  (0.12) | 0.37  (0.22) | 2.84  (0.32) | 1.59  (0.09) | 1.25***  (0.34) |
| **Iron**  **(mg)** | mean (SE) | 5.94  (0.51) | 4.21  (0.2) | 1.73***  (0.55) | 3.41  (0.41) | 4.81  (0.22) | **-**1.41*  (0.46) | 4.22  (0.42) | 4.7  (0.22) | **-**0.48  (0.47) | 5.55  (0.53) | 4.37  (0.2) | 1.18*  (0.57) |

^a^ We defined and applied four different carb ratios: per 10g of carb:(a) ≥1g fiber (10:1 carb:fiber), (b) ≥1g fiber and <1g free sugars (10:1:1 carb:fiber:free sugars), (c) ≥1g fiber and <2g free sugars (10:1:2 carb:fiber:free sugars); and (d) ≥1g fiber and, per each 1 g of fiber, <2g free sugars (10:1 carb:fiber, 1:2 fiber:free sugars; or 10:1|1:2).

^b^ All units were expressed as per 100 grams.

^c^ The amount of nutrients contained in each of the food code per 100 grams available in NHANES was calculated using US Department of Agriculture’s Food and Nutrient Database for Dietary Studies.

^d^ Statistical significances for differences (Dif.) is noted as ****P*<0.001, ***P*<0.01 and **P*<0.05.

^e^ Total sugars included both added sugar and natural sugar such as lactose present in milk and fructose present in whole or cut fruit and 100% fruit juice; added sugars included sugars that were added to foods as an ingredient during preparation, processing, or at the table; and free sugars included added sugars (e.g., honey, white sugar, syrups), sugar present in beverages (excluding sugars from dairy), sugar from fruit juice, and sugars from extruded fruit/vegetable products.

# **Table S5. Nutrient composition of available baby food-cereals, snacks and sweets meeting or not meeting each of four proposed metrics for assessing carbohydrate quality in the US**

|  |  | **Meeting or not Meeting each Metric**^s^ | | | | | | | | | | | |
| --- | --- | --- | --- | --- | --- | --- | --- | --- | --- | --- | --- | --- | --- |
| **Composition**^b^ | **Weighted**  **Values**^c^ | **10:1** | | | **10:1:1** | | | **10:1:2** | | | **10:1\|1:2** | | |
|  |  | **Yes** | **No** | **Dif.**^d^ | **Yes** | **No** | **Dif.** ^d^ | **Yes** | **No** | **Dif.** ^d^ | **Yes** | **No** | **Dif.** ^d^ |
|  | # (%) products | 4  (7.3%) | 51  (92.7%) |  | 3  (5.5%) | 52  (94.5%) |  | 3  (5.5%) | 52  (94.5%) |  | 3  (5.5%) | 52  (94.5%) |  |
| **Calories**  **(kJ)** | mean (SE) | 232.5  (83.6) | 227.9  (23.7) | 4.6  (86.9) | 285.7  (92.2) | 224.9  (23.4) | 60.8  (95.1) | 285.7  (92.2) | 224.9  (23.4) | 60.8  (95.1) | 285.7  (92.2) | 224.9  (23.4) | 60.8  (95.1) |
| **Total fat**  **(g)** | mean (SE) | 3.24  (1.40) | 3.39  (0.77) | **-**0.15  (1.60) | 4.25  (1.44) | 3.33  (0.76) | 0.92  (1.63) | 4.25  (1.44) | 3.33  (0.76) | 0.92  (1.63) | 4.25  (1.44) | 3.33  (0.76) | 0.92  (1.63) |
| **Saturated fat (g)** | mean (SE) | 0.66  (0.31) | 0.58  (0.12) | 0.08  (0.33) | 0.87  (0.33) | 0.57  (0.12) | 0.30  (0.35) | 0.87  (0.33) | 0.57  (0.12) | 0.30  (0.35) | 0.87  (0.33) | 0.57  (0.12) | 0.30  (0.35) |
| **Protein**  **(g)** | mean (SE) | 5.98  (2.37) | 3.77  (0.58) | 2.2  (2.44) | 7.8  (2.33) | 3.71  (0.57) | 4.09  (2.40) | 7.8  (2.33) | 3.71  (0.57) | 4.09  (2.40) | 7.8  (2.33) | 3.71  (0.57) | 4.09  (2.40) |
| **Total sugar (g)**^d^ | mean (SE) | 8.2  (2.79) | 15.4  (1.83) | **-**7.2*  (3.33) | 5.98  (2.64) | 15.39  (1.79) | **-**9.41**  (3.19) | 5.98  (2.64) | 15.39  (1.79) | **-**9.41**  (3.19) | 5.98  (2.64) | 15.39  (1.79) | **-**9.41**  (3.19) |
| **Added sugar (g)**^d^ | mean (SE) | 2.19  (2.19) | 7.16  (1.55) | **-**4.97*  (2.46) | 0 | 7.19  (1.52) | -7.19**  (1.52) | 0 | 7.19  (1.52) | -7.19**  (1.52) | 0 | 7.19  (1.52) | -7.19**  (6.38) |
| **Free sugar (g)**^d^ | mean (SE) | 2.19  (1.94) | 7.76  (1.55) | **-**5.56*  (2.48) | 0 | 7.78  (1.52) | -7.78***  (1.52) | 0 | 7.78  (1.51) | -7.78***  (1.52) | 0 | 7.78  (1.51) | -7.78***  (1.52) |
| **Fiber**  **(g)** | mean (SE) | 4.83  (1.52) | 1.90  (0.30) | 2.93*  (1.55) | 5.77  (1.69) | 1.90  (0.29) | 3.87**  (1.72) | 5.77  (1.69) | 1.90  (0.29) | 3.87**  (1.72) | 5.77  (1.69) | 1.90  (0.29) | 3.87**  (1.72) |
| **Sodium**  **(mg)** | mean (SE) | 17.8  (15.4) | 59  (15.9) | **-**41.2  (22.1) | 23.3  (19.4) | 57.9  (15.7) | **-**34.5  (24.9) | 23.3  (19.4) | 57.9  (15.7) | **-**34.5  (24.9) | 23.3  (19.4) | 57.9  (15.7) | **-**34.5  (24.9) |
| **Potassium (mg)** | mean (SE) | 300.3  (121) | 202.7  (27.3) | 97.6  (124) | 357.3  (146) | 201.3  (26.8) | 156.1  (149) | 357.3  (146) | 201.3  (27) | 156.1  (149) | 357.3  (147) | 201.3  (27) | 156.1  (149) |
| **Magnesium (mg)** | mean (SE) | 57  (21.6) | 29.5  (5.5) | 27.5  (22) | 73.7  (21) | 29.1  (5.4) | 44.6*  (22) | 73.7  (21) | 29.1  (5.4) | 44.6*  (22) | 73.7  (25.3) | 29.1  (5.4) | 44.6*  (22) |
| **Folic acid**  **(mcg)** | mean (SE) | 49.8  (44) | 34.4  (8.2) | 15.4  (45) | 66.3  (55) | 33.7  (8.1) | 32.6  (56) | 66.3  (55) | 33.7  (8) | 32.6  (56) | 66.3  (55) | 33.7  (8) | 32.6  (56) |
| **Thiamine**  **(mg)** | mean (SE) | 1.15  (0.79) | 0.49  (0.10) | 0.66  (0.79) | 1.53  (0.95) | 0.48  (0.10) | 1.05  (0.96) | 1.53  (0.95) | 0.48  (0.10) | 1.05  (0.96) | 1.53  (0.95) | 0.48  (0.10) | 1.05  (0.96) |
| **Riboflavin**  **(mg)** | mean (SE) | 1.12  (0.74) | 0.59  (0.12) | 0.53  (0.75) | 1.48  (0.89) | 0.58  (0.12) | 0.90  (0.90) | 1.48  (0.89) | 0.58  (0.12) | 0.90  (0.90) | 1.48  (0.89) | 0.58  (0.12) | 0.90  (0.90) |
| **Niacin**  **(mg)** | mean (SE) | 7.65  (4.27) | 6.25  (1.17) | 1.4  (4.42) | 10.11  (4.90) | 6.14  (1.15) | 3.97  (5.03) | 10.11  (4.90) | 6.14  (1.15) | 3.97  (5.03) | 10.11  (4.90) | 6.14  (1.15) | 3.97  (5.03) |
| **Vitamin B6 (mcg)**^d^ | mean (SE) | 0.22  (0.09) | 0.39  (0.13) | **-**0.17  (0.15) | 0.27  (0.11) | 0.39  (0.12) | **-**0.12  (0.16) | 0.27  (0.11) | 0.39  (0.12) | **-**0.12  (0.16) | 0.27  (0.11) | 0.39  (0.12) | **-**0.12  (0.16) |
| **Vitamin B12 (mcg)**^d^ | mean (SE) | 0.06  (0.05) | 0.62  (0.21) | **-**0.56*  (0.21) | 0.08  (0.07) | 0.61  (0.20) | -0.53*  (0.21) | 0.08  (0.07) | 0.61  (0.20) | -0.53*  (0.21) | 0.08  (0.07) | 0.61  (0.20) | -0.53*  (0.21) |
| **Vitamin E**  **(mcg)** | mean (SE) | 0.96  (0.47) | 1.72  (0.41) | **-**0.76  (0.62) | 1.02  (0.62) | 1.7  (0.40) | **-**0.68  (0.74) | 1.02  (0.62) | 1.7  (0.40) | **-**0.68  (0.74) | 1.02  (0.62) | 1.7  (0.40) | **-**0.68  (0.74) |
| **Zinc**  **(mg)** | mean (SE) | 1.95  (1.13) | 2.23  (0.55) | **-**0.28  (1.26) | 2.57  (1.31) | 2.18  (0.54) | 0.39  (1.42) | 2.57  (1.31) | 2.18  (0.54) | 0.39  (1.42) | 2.57  (1.31) | 2.18  (0.54) | 0.39  (1.42) |
| **Iron**  **(mg)** | mean (SE) | 15.66  (9.8) | 11.99  (2.5) | 3.67  (10.1) | 20.83  (11.6) | 11.76  (2.48) | 9.06  (11.8) | 20.83  (11.6) | 11.76  (2.46) | 9.06  (11.8) | 20.83  (11.6) | 11.76  (2.48) | 9.06  (11.8) |

^a^ We defined and applied four different carb ratios: per 10g of carb:(a) ≥1g fiber (10:1 carb:fiber), (b) ≥1g fiber and <1g free sugars (10:1:1 carb:fiber:free sugars), (c) ≥1g fiber and <2g free sugars (10:1:2 carb:fiber:free sugars); and (d) ≥1g fiber and, per each 1 g of fiber, <2g free sugars (10:1 carb:fiber, 1:2 fiber:free sugars; or 10:1|1:2).

^b^ All units were expressed as per 100 grams.

^c^ The amount of nutrients contained in each of the food code per 100 grams available in NHANES was calculated using US Department of Agriculture’s Food and Nutrient Database for Dietary Studies.

^d^ Statistical significances for differences (Dif.) is noted as ****P*<0.001, ***P*<0.01 and **P*<0.05.

^e^ Total sugars included both added sugar and natural sugar such as lactose present in milk and fructose present in whole or cut fruit and 100% fruit juice; added sugars included sugars that were added to foods as an ingredient during preparation, processing, or at the table; and free sugars included added sugars (e.g., honey, white sugar, syrups), sugar present in beverages (excluding sugars from dairy), sugar from fruit juice, and sugars from extruded fruit/vegetable products.

# **Table S6.** **Nutrient composition of available bread, rolls, and tortillas meeting or not meeting each of four proposed metrics for assessing carbohydrate quality in the US.**

|  |  | **Meeting or not Meeting each Metric**^s^ | | | | | | | | | | | |
| --- | --- | --- | --- | --- | --- | --- | --- | --- | --- | --- | --- | --- | --- |
| **Composition**^b^ | **Weighted**  **Values**^c^ | **10:1** | | | **10:1:1** | | | **10:1:2** | | | **10:1\|1:2** | | |
|  |  | **Yes** | **No** | **Dif.**^d^ | **Yes** | **No** | **Dif.** ^d^ | **Yes** | **No** | **Dif.** ^d^ | **Yes** | **No** | **Dif.** ^d^ |
|  | # (%) products | 64  (7.3%) | 142  (92.7%) |  | 51  (5.5%) | 155  (94.5%) |  | 64  (5.5%) | 142  (94.5%) |  | 64  (5.5%) | 142  (94.5%) |  |
| **Calories**  **(kJ)** | mean (SE) | 256.5  (5.7) | 289.3  (3.6) | **-**32.8***  (6.7) | 252.2  (6.9) | 288  (3.4) | **-**35.8***  (7.7) | 256.5  (5.7) | 289.3  (3.6) | **-**32.8***  (6.7) | 256.5  (5.7) | 289.3  (3.6) | **-**32.8***  (6.7) |
| **Total fat**  **(g)** | mean (SE) | 3.7  (0.38) | 5.84  (0.45) | **-**2.14**  (0.59) | 3.65  (0.48) | 5.68  (0.41) | **-**2.03**  (0.63) | 3.7  (0.38) | 5.84  (0.45) | **-**2.14**  (0.59) | 3.7  (0.38) | 5.84  (0.45) | **-**2.14**  (0.59) |
| **Saturated fat (g)** | mean (SE) | 0.99  (0.16) | 1.44  (0.14) | **-**0.45*  (0.21) | 1.03  (0.20) | 1.39  (0.13) | **-**0.35  (0.23) | 0.99  (0.16) | 1.44  (0.14) | **-**0.45*  (0.21) | 0.99  (0.16) | 1.44  (0.14) | **-**0.45*  (0.21) |
| **Protein**  **(g)** | mean (SE) | 10.5  (0.36) | 9.49  (0.15) | 1.02**  (0.39) | 10.23  (0.39) | 9.66  (0.16) | 0.56  (0.42) | 10.5  (0.36) | 9.49  (0.15) | 1.02**  (0.39) | 10.5  (0.36) | 9.49  (0.15) | 1.02**  (0.39) |
| **Total sugar (g)**^d^ | mean (SE) | 4.74  (0.49) | 5.35  (0.30) | -0.61  (0.56) | 3.53  (0.36) | 5.69  (0.31) | **-**2.16***  (0.58) | 4.74  (0.49) | 5.35  (0.30) | -0.61  (0.56) | 4.74  (0.49) | 5.35  (0.30) | -0.61  (0.56) |
| **Added sugar (g)**^d^ | mean (SE) | 2.68  (0.31) | 3.39  (0.24) | **-**0.71  (0.39) | 1.65  (0.18) | 3.67  (0.24) | **-**2.02***  (0.30) | 2.68  (0.31) | 3.39  (0.24) | **-**0.71  (0.39) | 2.68  (0.31) | 3.39  (0.24) | **-**0.71  (0.39) |
| **Free sugar (g)**^d^ | mean (SE) | 2.68  (0.31) | 3.39  (0.24) | **-**0.71  (0.42) | 1.65  (0.18) | 3.67  (0.24) | **-**2.02***  (0.43) | 2.68  (0.31) | 3.39  (0.24) | **-**0.71  (0.42) | 2.68  (0.31) | 3.39  (0.24) | **-**0.71  (0.42) |
| **Fiber**  **(g)** | mean (SE) | 7.36  (0.29) | 2.95  (0.08) | 4.4***  (0.30) | 7.53  (0.36) | 3.27  (0.11) | 4.26***  (0.37) | 7.36  (0.29) | 2.95  (0.08) | 4.4***  (0.30) | 7.36  (0.29) | 2.95  (0.08) | 4.4***  (0.30) |
| **Sodium**  **(mg)** | mean (SE) | 468.5  (15.5) | 504  (8.9) | **-**35.6*  (17.9) | 475.4  (18.7) | 498.8  (8.4) | **-**23.4  (20.5) | 468.5  (15.5) | 504  (8.9) | **-**35.6*  (17.9) | 468.5  (15.5) | 504  (8.9) | **-**35.6*  (17.9) |
| **Potassium (mg)** | mean (SE) | 220.5  (15.5) | 146.1  (4.4) | 74.5***  (16.1) | 192  (10.8) | 161.7  (7.3) | 30.3*  (13.1) | 220.5  (15.5) | 146.1  (4.4) | 74.5***  (16.1) | 220.5  (15.5) | 146.1  (4.4) | 74.5***  (16.1) |
| **Magnesium (mg)** | mean (SE) | 54.1  (2.6) | 31.7  (1.1) | 22.4***  (2.8) | 51  (2.6) | 34.6  (1.4) | 16.4***  (2.9) | 54.1  (2.6) | 31.7  (1.1) | 22.4***  (2.8) | 54.1  (2.6) | 31.7  (1.1) | 22.4***  (2.8) |
| **Folic acid**  **(mcg)** | mean (SE) | 30.2  (3.3) | 60  (2.3) | **-**29.8***  (4.0) | 36.6  (3.5) | 55.3  (2.4) | **-**18.7***  (4.3) | 30.2  (3.3) | 60  (2.3) | **-**29.8***  (4.0) | 30.2  (3.3) | 60  (2.3) | **-**29.8***  (4.0) |
| **Thiamine**  **(mg)** | mean (SE) | 0.35  (0.01) | 0.46  (0.01) | **-**0.11***  (0.02) | 0.35  (0.01) | 0.45  (0.01) | **-**0.09***  (0.02) | 0.35  (0.01) | 0.46  (0.01) | **-**0.11***  (0.02) | 0.35  (0.01) | 0.46  (0.01) | **-**0.11***  (0.02) |
| **Riboflavin**  **(mg)** | mean (SE) | 0.22  (0.01) | 0.32  (0.01) | **-**0.11***  (0.01) | 0.23  (0.01) | 0.31  (0.01) | **-**0.08***  (0.02) | 0.22  (0.01) | 0.32  (0.01) | **-**0.11***  (0.01) | 0.22  (0.01) | 0.32  (0.01) | **-**0.11***  (0.01) |
| **Niacin**  **(mg)** | mean (SE) | 3.52  (0.11) | 4.37  (0.08) | **-**0.84***  (0.13) | 3.51  (0.11) | 4.3  (0.08) | **-**0.80***  (0.13) | 3.52  (0.11) | 4.37  (0.08) | **-**0.84***  (0.13) | 3.52  (0.11) | 4.37  (0.08) | **-**0.84***  (0.13) |
| **Vitamin B6 (mcg)**^d^ | mean (SE) | 0.15  (0.01) | 0.09  (0.001) | 0.06***  (0.01) | 0.13  (0.01) | 0.10  (0.001) | 0.03**  (0.01) | 0.15  (0.01) | 0.09  (0.001) | 0.06***  (0.01) | 0.15  (0.01) | 0.09  (0.001) | 0.06***  (0.01) |
| **Vitamin B12 (mcg)**^d^ | mean (SE) | 0.03  (0.01) | 0.05  (0.01) | **-**0.02  (0.01) | 0.03  (0.01) | 0.05  (0.01) | **-**0.02  (0.01) | 0.03  (0.01) | 0.05  (0.01) | **-**0.02  (0.01) | 0.03  (0.01) | 0.05  (0.01) | **-**0.02  (0.01) |
| **Vitamin E**  **(mcg)** | mean (SE) | 0.43  (0.03) | 0.53  (0.05) | **-**0.10  (0.06) | 0.43  (0.04) | 0.52  (0.05) | **-**0.09  (0.06) | 0.43  (0.03) | 0.53  (0.05) | **-**0.10  (0.06) | 0.43  (0.03) | 0.53  (0.05) | **-**0.10  (0.06) |
| **Zinc**  **(mg)** | mean (SE) | 1.37  (0.04) | 0.97  (0.02) | 0.40***  (0.04) | 1.32  (0.04) | 1.02  (0.03) | 0.30***  (0.05) | 1.37  (0.04) | 0.97  (0.02) | 0.40***  (0.04) | 1.37  (0.04) | 0.97  (0.02) | 0.40***  (0.04) |
| **Iron**  **(mg)** | mean (SE) | 2.88  (0.10) | 3.23  (0.05) | **-**0.35**  (0.11) | 2.97  (0.12) | 3.17  (0.05) | **-**0.20  (0.13) | 2.88  (0.10) | 3.23  (0.05) | **-**0.35**  (0.11) | 2.88  (0.10) | 3.23  (0.05) | **-**0.35**  (0.11) |

^a^ We defined and applied four different carb ratios: per 10g of carb:(a) ≥1g fiber (10:1 carb:fiber), (b) ≥1g fiber and <1g free sugars (10:1:1 carb:fiber:free sugars), (c) ≥1g fiber and <2g free sugars (10:1:2 carb:fiber:free sugars); and (d) ≥1g fiber and, per each 1 g of fiber, <2g free sugars (10:1 carb:fiber, 1:2 fiber:free sugars; or 10:1|1:2).

^b^ All units were expressed as per 100 grams.

^c^ The amount of nutrients contained in each of the food code per 100 grams available in NHANES was calculated using US Department of Agriculture’s Food and Nutrient Database for Dietary Studies.

^d^ Statistical significances for differences (Dif.) is noted as ****P*<0.001, ***P*<0.01 and **P*<0.05.

^e^ Total sugars included both added sugar and natural sugar such as lactose present in milk and fructose present in whole or cut fruit and 100% fruit juice; added sugars included sugars that were added to foods as an ingredient during preparation, processing, or at the table; and free sugars included added sugars (e.g., honey, white sugar, syrups), sugar present in beverages (excluding sugars from dairy), sugar from fruit juice, and sugars from extruded fruit/vegetable products.

# **Table S7. Nutrient composition of available cooked cereals meeting or not meeting each of four proposed metrics for assessing carbohydrate quality in the US.**

|  |  | **Meeting or not Meeting each Metric**^s^ | | | | | | | | | | | |
| --- | --- | --- | --- | --- | --- | --- | --- | --- | --- | --- | --- | --- | --- |
| **Composition**^b^ | **Weighted**  **Values**^c^ | **10:1** | | | **10:1:1** | | | **10:1:2** | | | **10:1\|1:2** | | |
|  |  | **Yes** | **No** | **Dif.**^d^ | **Yes** | **No** | **Dif.** ^d^ | **Yes** | **No** | **Dif.** ^d^ | **Yes** | **No** | **Dif.** ^d^ |
|  | # (%) products | 76  (7.3%) | 121  (92.7%) |  | 59  (5.5%) | 138  (94.5%) |  | 66  (5.5%) | 131  (94.5%) |  | 75  (5.5%) | 122  (94.5%) |  |
| **Calories**  **(kJ)** | mean (SE) | 84.9  (2.8) | 94.6  (2.5) | **-**9.6*  (3.7) | 80.3  (3.2) | 95.4  (2.2) | **-**15.1***  (3.9) | 83.4  (3.1) | 94.6  (2.3) | **-**11.2**  (3.9) | 84.7  (2.8) | 94.6  (2.5) | **-**9.9*  (3.7) |
| **Total fat**  **(g)** | mean (SE) | 2.49  (0.17) | 2.48  (0.13) | 0.02  (0.21) | 2.34  (0.19) | 2.54  (0.12) | **-**0.20  (0.22) | 2.5  (0.18) | 2.47  (0.12) | 0.03  (0.22) | 2.51  (0.17) | 2.47  (0.13) | 0.04  (0.21) |
| **Saturated fat (g)** | mean (SE) | 0.87  (0.07) | 1.09  (0.07) | **-**0.23*  (0.10) | 0.86  (0.08) | 1.07  (0.06) | **-**0.20  (0.11) | 0.89  (0.08) | 1.06  (0.07) | **-**0.18  (0.10) | 0.87  (0.07) | 1.08  (0.07) | **-**0.21*  (0.10) |
| **Protein**  **(g)** | mean (SE) | 3.0  (0.16) | 2.91  (0.14) | 0.09  (0.21) | 3.06  (0.20) | 2.9  (0.12) | 0.16  (0.23) | 3.09  (0.18) | 2.87  (0.13) | 0.22  (0.22) | 3.01  (0.16) | 2.91  (0.14) | 0.10  (0.21) |
| **Total sugar (g)**^d^ | mean (SE) | 2.36  (0.26) | 3.65  (0.33) | **-**1.29**  (0.42) | 1.87  (0.29) | 3.70  (0.29) | **-**1.82***  (0.41) | 2.07  (0.28) | 3.69  (0.31) | **-**1.62**  (0.41) | 2.29  (0.25) | 3.68  (0.33) | **-**1.39**  (0.42) |
| **Added sugar (g)**^d^ | mean (SE) | 0.77  (0.17) | 1.68  (0.27) | **-**0.91*  (0.37) | 0 | 1.9  (0.25) | **-**1.9***  (0.38) | 0.3  (0.11) | 1.85  (0.26) | **-**1.55***  (0.37) | 0.68  (0.15) | 1.72  (0.28) | **-**1.04**  (0.37) |
| **Free sugar (g)**^d^ | mean (SE) | 0.77  (0.17) | 1.68  (0.27) | **-**0.91*  (0.32) | 0 | 1.9  (0.25) | **-**1.9***  (0.25) | 0.30  (0.11) | 1.85  (0.26) | **-**1.55***  (0.28) | 0.68  (0.15) | 1.72  (0.28) | **-**1.04**  (0.32) |
| **Fiber**  **(g)** | mean (SE) | 1.81  (0.04) | 0.77  (0.04) | 1.04***  (0.06) | 1.76  (0.05) | 0.91  (0.05) | 0.85***  (0.07) | 1.78  (0.04) | 0.86  (0.05) | 0.93***  (0.06) | 1.8  (0.04) | 0.78  (0.04) | 1.02***  (0.06) |
| **Sodium**  **(mg)** | mean (SE) | 139.1  (12.2) | 156  (7.0) | **-**16.9  (14.1) | 147.2  (15.4) | 150.5  (6.3) | **-**3.3  (13.9) | 144.3  (13.9) | 152.2  (6.6) | **-**7.9  (15.4) | 138.9  (12.3) | 156.1  (7.0) | **-**17.2  (14.2) |
| **Potassium (mg)** | mean (SE) | 102.9  (6.8) | 86.1  (5.4) | 16.8  (8.7) | 103.1  (8.5) | 88.1  (4.9) | 15  (9.8) | 106.6  (7.7) | 85.5  (5.0) | 21.1  (9.2) | 103.3  (6.9) | 86  (5.4) | 17.2  (8.7) |
| **Magnesium (mg)** | mean (SE) | 26.8  (0.8) | 12.6  (0.6) | 14.2***  (1.0) | 26.6  (1.0) | 14.5  (0.70) | 12.2***  (1.2) | 27.1  (0.9) | 13.5  (0.6) | 13.6***  (1.1) | 26.9  (0.8) | 12.7  (0.6) | 14.1***  (1.0) |
| **Folic acid**  **(mcg)** | mean (SE) | 3.8  (1.1) | 19.8  (3.4) | **-**16***  (3.5) | 0.70  (0.70) | 19.2  (3.0) | **-**18.5***  (3.1) | 1.0  (0.7) | 20.0  (3.1) | **-**19***  (3.2) | 3.2  (0.9) | 20  (3.3) | **-**16.9***  (3.5) |
| **Thiamine**  **(mg)** | mean (SE) | 0.09  (0.01) | 0.15  (0.01) | **-**0.06***  (0.01) | 0.08  (0.01) | 0.15  (0.01) | **-**0.07***  (0.01) | 0.08  (0.01) | 0.15  (0.01) | **-**0.07***  (0.01) | 0.09  (0.01) | 0.15  (0.01) | **-**0.06***  (0.01) |
| **Riboflavin**  **(mg)** | mean (SE) | 0.09  (0.01) | 0.15  (0.01) | **-**0.06***  (0.01) | 0.08  (0.01) | 0.15  (0.01) | **-**0.07***  (0.01) | 0.09  (0.01) | 0.15  (0.01) | **-**0.06***  (0.01) | 0.09  (0.01) | 0.15  (0.01) | **-**0.06***  (0.01) |
| **Niacin**  **(mg)** | mean (SE) | 0.52  (0.07) | 1.13  (0.08) | **-**0.61***  (0.11) | 0.35  (0.05) | 1.13  (0.08) | **-**0.78***  (0.09) | 0.37  (0.05) | 1.17  (0.08) | **-**0.80***  (0.09) | 0.50  (0.06) | 1.14  (0.08) | **-**0.65***  (0.10) |
| **Vitamin B6 (mcg)**^d^ | mean (SE) | 0.09  (0.01) | 0.11  (0.01) | **-**0.02  (0.01) | 0.07  (0.01) | 0.12  (0.01) | **-**0.05**  (0.01) | 0.07  (0.01) | 0.12  (0.01) | **-**0.05**  (0.01) | 0.09  (0.01) | 0.11  (0.01) | **-**0.03  (0.01) |
| **Vitamin B12 (mcg)**^d^ | mean (SE) | 0.14  (0.02) | 0.21  (0.02) | **-**0.07*  (0.03) | 0.12  (0.02) | 0.21  (0.02) | **-**0.09**  (0.03) | 0.17  (0.03) | 0.19  (0.02) | **-**0.03  (0.03) | 0.15  (0.02) | 0.21  (0.02) | **-**0.06  (0.03) |
| **Vitamin E**  **(mcg)** | mean (SE) | 0.44  (0.06) | 0.38  (0.05) | 0.05  (0.08) | 0.30  (0.04) | 0.45  (0.05) | **-**0.15  (0.06) | 0.45  (0.07) | 0.38  (0.05) | 0.07  (0.08) | 0.44  (0.06) | 0.38  (0.05) | 0.06  (0.08) |
| **Zinc**  **(mg)** | mean (SE) | 0.67  (0.02) | 0.38  (0.02) | 0.29***  (0.03) | 0.68  (0.03) | 0.41  (0.02) | 0.27***  (0.03) | 0.69  (0.03) | 0.39  (0.02) | 0.30***  (0.03) | 0.67  (0.02) | 0.38  (0.02) | 0.29***  (0.03) |
| **Iron**  **(mg)** | mean (SE) | 1.60  (0.18) | 2.65  (0.22) | **-**1.04**  (0.28) | 1.12  (0.18) | 2.72  (0.19) | **-**1.60***  (0.26) | 1.32  (0.19) | 2.71  (0.20) | **-**1.39***  (0.28) | 1.59  (0.19) | 2.64  (0.21) | **-**1.05**  (0.28) |

^a^ We defined and applied four different carb ratios: per 10g of carb:(a) ≥1g fiber (10:1 carb:fiber), (b) ≥1g fiber and <1g free sugars (10:1:1 carb:fiber:free sugars), (c) ≥1g fiber and <2g free sugars (10:1:2 carb:fiber:free sugars); and (d) ≥1g fiber and, per each 1 g of fiber, <2g free sugars (10:1 carb:fiber, 1:2 fiber:free sugars; or 10:1|1:2).

^b^ All units were expressed as per 100 grams.

^c^ The amount of nutrients contained in each of the food code per 100 grams available in NHANES was calculated using US Department of Agriculture’s Food and Nutrient Database for Dietary Studies.

^d^ Statistical significances for differences (Dif.) is noted as ****P*<0.001, ***P*<0.01 and **P*<0.05.

^e^ Total sugars included both added sugar and natural sugar such as lactose present in milk and fructose present in whole or cut fruit and 100% fruit juice; added sugars included sugars that were added to foods as an ingredient during preparation, processing, or at the table; and free sugars included added sugars (e.g., honey, white sugar, syrups), sugar present in beverages (excluding sugars from dairy), sugar from fruit juice, and sugars from extruded fruit/vegetable products.

# **Table S8. Nutrient composition of available cooked grains products meeting or not meeting each of four proposed metrics for assessing carbohydrate quality in the US.**

|  |  | **Meeting or not Meeting each Metric**^s^ | | | | | | | | | | | |
| --- | --- | --- | --- | --- | --- | --- | --- | --- | --- | --- | --- | --- | --- |
| **Composition**^b^ | **Weighted**  **Values**^c^ | **10:1** | | | **10:1:1** | | | **10:1:2** | | | **10:1\|1:2** | | |
|  |  | **Yes** | **No** | **Dif.**^d^ | **Yes** | **No** | **Dif.** ^d^ | **Yes** | **No** | **Dif.** ^d^ | **Yes** | **No** | **Dif.** ^d^ |
|  | # (%) products | 27  (7.3%) | 55  (92.7%) |  | 27  (5.5%) | 55  (94.5%) |  | 27  (5.5%) | 55  (94.5%) |  | 27  (5.5%) | 55  (94.5%) |  |
| **Calories**  **(kJ)** | mean (SE) | 131.4  (4.7) | 128.9  (3.8) | 2.5  (6.0) | 131.4  (4.7) | 128.9  (3.8) | 2.5  (6.0) | 131.4  (4.7) | 128.9  (3.8) | 2.5  (6.0) | 131.4  (4.7) | 128.9  (3.8) | 2.5  (6.0) |
| **Total fat**  **(g)** | mean (SE) | 1.97  (0.32) | 1.83  (0.21) | 0.15  (0.38) | 1.97  (0.32) | 1.83  (0.21) | 0.15  (0.38) | 1.97  (0.32) | 1.83  (0.21) | 0.15  (0.38) | 1.97  (0.32) | 1.83  (0.21) | 0.15  (0.38) |
| **Saturated fat (g)** | mean (SE) | 0.34  (0.06) | 0.37  (0.05) | **-**0.04  (0.08) | 0.34  (0.06) | 0.37  (0.05) | **-**0.04  (0.08) | 0.34  (0.06) | 0.37  (0.05) | **-**0.04  (0.08) | 0.34  (0.06) | 0.37  (0.05) | **-**0.04  (0.08) |
| **Protein**  **(g)** | mean (SE) | 4.36  (0.25) | 3.06  (0.20) | 1.30***  (0.32) | 4.36  (0.24) | 3.06  (0.20) | 1.30***  (0.32) | 4.36  (0.24) | 3.06  (0.20) | 1.30***  (0.32) | 4.36  (0.24) | 3.06  (0.20) | 1.30*** (0.32) |
| **Total sugar (g)**^d^ | mean (SE) | 0.72  (0.06) | 0.56  (0.17) | 0.16  (0.18) | 0.72  (0.06) | 0.56  (0.17) | 0.16  (0.18) | 0.72  (0.06) | 0.56  (0.17) | 0.16  (0.18) | 0.72  (0.06) | 0.56  (0.17) | 0.16  (0.18) |
| **Added sugar (g)**^d^ | mean (SE) | 0 | 0.09  (0.09) | **-**0.09  (0.09) | 0 | 0.09  (0.09) | **-**0.09  (0.09) | 0 | 0.09  (0.09) | **-**0.09  (0.09) | 0 | 0.09  (0.09) | **-**0.09  (0.09) |
| **Free sugar (g)**^d^ | mean (SE) | 0 | 0.09  (0.09) | **-**0.09  (0.09) | 0 | 0.09  (0.09) | **-**0.09  (0.09) | 0 | 0.09  (0.09) | **-**0.09  (0.09) | 0 | 0.09  (0.09) | **-**0.09  (0.09) |
| **Fiber**  **(g)** | mean (SE) | 3.68  (0.11) | 1.09  (0.08) | 2.59***  (0.14) | 3.68  (0.11) | 1.09  (0.08) | 2.59***  (0.14) | 3.68  (0.11) | 1.09  (0.08) | 2.59***  (0.14) | 3.68  (0.11) | 1.09  (0.08) | 2.59***  (0.14) |
| **Sodium**  **(mg)** | mean (SE) | 194.5  (8.5) | 235.9  (15.2) | **-**41.4  (17.4) | 194.5  (8.5) | 235.9  (15.2) | **-**41.4  (17.4) | 194.5  (8.5) | 235.9  (15.2) | **-**41.4  (17.4) | 194.5  (8.5) | 235.9  (15.2) | **-**41.4  (17.4) |
| **Potassium (mg)** | mean (SE) | 83.1  (7.4) | 57.4  (6.3) | 25.7*  (9.7) | 83.1  (7.4) | 57.4  (6.3) | 25.7*  (9.7) | 83.1  (7.4) | 57.4  (6.2) | 25.7*  (9.7) | 83.1  (7.4) | 57.4  (6.2) | 25.7*  (9.7) |
| **Magnesium (mg)** | mean (SE) | 44  (2.9) | 20.6  (1.9) | 23.4***  (3.5) | 44  (2.9) | 20.6  (1.9) | 23.4***  (3.5) | 44  (2.9) | 20.6  (1.9) | 23.4***  (3.5) | 44  (2.9) | 20.6  (1.9) | 23.4***  (3.5) |
| **Folic acid**  **(mcg)** | mean (SE) | 6.5  (3.6) | 25.1  (3.9) | **-**18.6***  (5.3) | 6.5  (3.6) | 25.1  (3.9) | **-**18.6***  (5.3) | 6.5  (3.6) | 25.1  (3.9) | **-**18.6***  (5.3) | 6.5  (3.6) | 25.1  (3.9) | **-**18.6***  (5.3) |
| **Thiamine**  **(mg)** | mean (SE) | 0.10  (0.01) | 0.14  (0.01) | **-**0.04*  (0.01) | 0.10  (0.01) | 0.14  (0.01) | **-**0.04*  (0.01) | 0.10  (0.01) | 0.14  (0.01) | **-**0.04*  (0.01) | 0.10  (0.01) | 0.14  (0.01) | **-**0.04*  (0.01) |
| **Riboflavin**  **(mg)** | mean (SE) | 0.07  (0.01) | 0.06  (0.01) | 0.01  (0.01) | 0.07  (0.01) | 0.06  (0.01) | 0.01  (0.01) | 0.07  (0.01) | 0.06  (0.01) | 0.01  (0.01) | 0.07  (0.01) | 0.06  (0.01) | 0.01  (0.01) |
| **Niacin**  **(mg)** | mean (SE) | 1.67  (0.2) | 1.34  (0.1) | 0.32  (0.2) | 1.67  (0.2) | 1.34  (0.1) | 0.32  (0.2) | 1.67  (0.2) | 1.34  (0.1) | 0.32  (0.2) | 1.67  (0.2) | 1.34  (0.1) | 0.32  (0.2) |
| **Vitamin B6 (mcg)**^d^ | mean (SE) | 0.12  (0.02) | 0.08  (0.01) | 0.03*  (0.02) | 0.12  (0.02) | 0.08  (0.01) | 0.03*  (0.02) | 0.12  (0.02) | 0.08  (0.01) | 0.03*  (0.02) | 0.12  (0.02) | 0.08  (0.01) | 0.03*  (0.02) |
| **Vitamin B12 (mcg)**^d^ | mean (SE) | 0 | 0.01  (0.01) | **-**0.01*  (0.01) | 0 | -0.01*  (0.01) | **-**0.01  (0.01) | 0 | 0.01  (0.01) | **-**0.01*  (0.01) | 0 | 0.01  (0.01) | **-**0.01*  (0.01) |
| **Vitamin E**  **(mcg)** | mean (SE) | 0.44  (0.09) | 0.27  (0.03) | 0.17  (0.10) | 0.44  (0.09) | 0.27  (0.03) | 0.17  (0.10) | 0.44  (0.09) | 0.27  (0.03) | 0.17  (0.10) | 0.44  (0.09) | 0.27  (0.03) | 0.17  (0.10) |
| **Zinc**  **(mg)** | mean (SE) | 0.92  (0.06) | 0.58  (0.05) | 0.33***  (0.08) | 0.92  (0.06) | 0.58  (0.05) | 0.33***  (0.08) | 0.92  (0.06) | 0.58  (0.05) | 0.33***  (0.08) | 0.92  (0.06) | 0.58  (0.05) | 0.33***  (0.08) |
| **Iron**  **(mg)** | mean (SE) | 1.14  (0.09) | 0.80  (0.06) | 0.34**  (0.11) | 1.14  (0.09) | 0.80  (0.06) | 0.34**  (0.11) | 1.14  (0.09) | 0.80  (0.06) | 0.34**  (0.11) | 1.14  (0.09) | 0.80  (0.06) | 0.34**  (0.11) |

^a^ We defined and applied four different carb ratios: per 10g of carb:(a) ≥1g fiber (10:1 carb:fiber), (b) ≥1g fiber and <1g free sugars (10:1:1 carb:fiber:free sugars), (c) ≥1g fiber and <2g free sugars (10:1:2 carb:fiber:free sugars); and (d) ≥1g fiber and, per each 1 g of fiber, <2g free sugars (10:1 carb:fiber, 1:2 fiber:free sugars; or 10:1|1:2).

^b^ All units were expressed as per 100 grams.

^c^ The amount of nutrients contained in each of the food code per 100 grams available in NHANES was calculated using US Department of Agriculture’s Food and Nutrient Database for Dietary Studies.

^d^ Statistical significances for differences (Dif.) is noted as ****P*<0.001, ***P*<0.01 and **P*<0.05. ^e^ Total sugars included both added sugar and natural sugar such as lactose present in milk and fructose present in whole or cut fruit and 100% fruit juice; added sugars included sugars that were added to foods as an ingredient during preparation, processing, or at the table; and free sugars included added sugars (e.g., honey, white sugar, syrups), sugar present in beverages (excluding sugars from dairy), sugar from fruit juice, and sugars from extruded fruit/vegetable products.

# **Table S9. Nutrient composition of available crackers products meeting or not meeting each of four proposed metrics for assessing carbohydrate quality in the US**

|  |  | **Meeting or not Meeting each Metric**^s^ | | | | | | | | | | | |
| --- | --- | --- | --- | --- | --- | --- | --- | --- | --- | --- | --- | --- | --- |
| **Composition**^b^ | **Weighted**  **Values**^c^ | **10:1** | | | **10:1:1** | | | **10:1:2** | | | **10:1\|1:2** | | |
|  |  | **Yes** | **No** | **Dif.**^d^ | **Yes** | **No** | **Dif.** ^d^ | **Yes** | **No** | **Dif.** ^d^ | **Yes** | **No** | **Dif.** ^d^ |
|  | # (%) products | 26  (32.5%) | 54  (67.5%) |  | 17  (21.3%) | 63  (78.7%) |  | 25  (31.3%) | 55  (68.7%) |  | 25  (31.3%) | 55  (68.7%) |  |
| **Calories**  **(kJ)** | mean (SE) | 427.5  (6) | 442.7  (6.4) | **-**15.2  (8.7) | 419.6  (8.3) | 442.6  (5.5) | **-**23*  (10.0) | 427.5  (6.2) | 442.4  (6.3) | **-**14.9  (8.8) | 427.5  (6.2) | 442.4  (6.3) | **-**14.9  (8.8) |
| **Total fat**  **(g)** | mean (SE) | 13.29  (1.07) | 14.16  (1.25) | **-**0.87  (1.64) | 11.85  (1.47) | 14.42  (1.08) | **-**2.57  (1.82) | 13.21  (1.10) | 14.18  (1.23) | **-**0.96  (1.65) | 13.21  (1.10) | 14.18  (1.23) | **-**0.96  (1.65) |
| **Saturated fat (g)** | mean (SE) | 2.29  (0.22) | 2.75  (0.29) | **-**0.45  (0.37) | 1.99  (0.30) | 2.76  (0.25) | **-**0.77  (0.39) | 2.3  (0.23) | 2.74  (0.29) | **-**0.44  (0.37) | 2.3  (0.23) | 2.74  (0.29) | **-**0.44  (0.37) |
| **Protein**  **(g)** | mean (SE) | 9.63  (0.34) | 9.13  (0.25) | 0.50  (0.42) | 9.95  (0.35) | 9.12  (0.23) | 0.83  (0.42) | 9.73  (0.34) | 9.1  (0.24) | 0.63  (0.42) | 9.73  (0.34) | 9.1  (0.24) | 0.63  (0.42) |
| **Total sugar (g)**^d^ | mean (SE) | 4.33  (1.06) | 4.97  (0.70) | **-**0.64  (1.27) | 1.02  (0.21) | 5.77  (0.69) | **-**4.74**  (0.72) | 3.59  (0.79) | 5.29  (0.76) | **-**1.7  (1.10) | 3.59  (0.79) | 5.29  (0.76) | **-**1.7  (1.10) |
| **Added sugar (g)**^d^ | mean (SE) | 3.88  (1.09) | 3.92  (0.65) | **-**0.04  (1.27) | 0.46  (0.21) | 4.84  (0.66) | **-**4.38**  (0.70) | 3.12  (0.81) | 4.26  (0.73) | **-**1.14  (1.21) | 3.12  (0.82) | 4.26  (0.72) | **-**1.14  (1.09) |
| **Free sugar (g)**^d^ | mean (SE) | 3.88  (1.09) | 3.92  (0.65) | **-**0.04  (1.2) | 0.46  (0.21) | 4.84  (0.66) | **-**4.38**  (1.29) | 3.12  (0.82) | 4.26  (0.72) | **-**1.15  (1.21) | 3.12  (0.82) | 4.26  (0.72) | **-**1.15  (1.21) |
| **Fiber**  **(g)** | mean (SE) | 10.5  (0.67) | 3.28  (0.22) | 7.22***  (0.70) | 11.46  (0.92) | 4.05  (0.30) | 7.42***  (0.97) | 10.62  (0.68) | 3.35  (0.23) | 7.27***  (0.71) | 10.62  (0.67) | 3.35  (0.23) | 7.27***  (0.71) |
| **Sodium**  **(mg)** | mean (SE) | 509.5  (42.2) | 599.5  (46.3) | **-**90  (62.7) | 460.4  (52.4) | 599.9  (40.6) | **-**139.6*  (66.3) | 514  (44) | 595.8  (45.5) | **-**81.8  (63.2) | 514  (44) | 595.8  (45.5) | **-**81.8  (63.2) |
| **Potassium (mg)** | mean (SE) | 320.5  (11) | 216.2  (20.6) | 104.3**  (23.3) | 325.8  (16.4) | 229.7  (18.1) | 96.1**  (24.5) | 322.3  (11.2) | 217.3  (20.2) | 105**  (23.1) | 322.3  (11.2) | 217.3  (20.2) | 105**  (23.1) |
| **Magnesium (mg)** | mean (SE) | 102.8  (5.3) | 47.9  (5.6) | 54.9***  (7.6) | 108.2  (7.6) | 54.3  (5.2) | 53.9***  (9.2) | 103.8  (5.4) | 48.5  (5.5) | 55.3***  (7.7) | 103.8  (5.4) | 48.5  (5.5) | 55.3***  (7.7) |
| **Folic acid**  **(mcg)** | mean (SE) | 11.1  (4) | 64.1  (6.8) | **-**53.1***  (7.8) | 13.4  (5.7) | 55.9  (6.4) | **-**42.6**  (8.5) | 10.7  (4.1) | 63.3  (6.7) | **-**52.7***  (7.9) | 10.7  (4.1) | 63.3  (6.7) | **-**52.7***  (7.8) |
| **Thiamine**  **(mg)** | mean (SE) | 0.26  (0.02) | 0.43  (0.03) | **-**0.16***  (0.03) | 0.26  (0.03) | 0.41  (0.02) | **-**0.15**  (0.04) | 0.26  (0.02) | 0.43  (0.03) | **-**0.17***  (0.03) | 0.26  (0.02) | 0.43  (0.03) | **-**0.17***  (0.03) |
| **Riboflavin**  **(mg)** | mean (SE) | 0.14  (0.02) | 0.31  (0.02) | **-**0.17***  (0.03) | 0.16  (0.03) | 0.28  (0.02) | **-**0.12**  (0.04) | 0.14  (0.02) | 0.31  (0.02) | **-**0.16***  (0.03) | 0.14  (0.02) | 0.31  (0.02) | **-**0.16***  (0.03) |
| **Niacin**  **(mg)** | mean (SE) | 4.27  (0.24) | 4.99  (0.21) | **-**0.73*  (0.32) | 4.17  (0.35) | 4.91  (0.19) | **-**0.74  (0.40) | 4.27  (0.25) | 4.97  (0.21) | **-**0.70  (0.33) | 4.27  (0.25) | 4.97  (0.21) | **-**0.70  (0.33) |
| **Vitamin B6 (mcg)**^d^ | mean (SE) | 0.22  (0.01) | 0.13  (0.01) | 0.09***  (0.02) | 0.23  (0.02) | 0.14  (0.01) | 0.09**  (0.02) | 0.23  (0.01) | 0.13  (0.01) | 0.10***  (0.02) | 0.23  (0.01) | 0.13  (0.01) | 0.10***  (0.02) |
| **Vitamin B12 (mcg)**^d^ | mean (SE) | 0 | 0.04  (0.01) | **-**0.04*  (0.01) | 0 | 0.03  (0.01) | **-**0.03  (0.01) | 0 | 0.04  (0.01) | **-**0.04*  (0.01) | 0 | 0.04  (0.01) | **-**0.04*  (0.01) |
| **Vitamin E**  **(mcg)** | mean (SE) | 1.36  (0.13) | 1.58  (0.17) | **-**0.22  (0.21) | 1.21  (0.17) | 1.58  (0.14) | **-**0.37  (0.23) | 1.35  (0.13) | 1.57  (0.16) | **-**0.22  (0.21) | 1.35  (0.13) | 1.57  (0.16) | **-**0.22  (0.21) |
| **Zinc**  **(mg)** | mean (SE) | 2.26  (0.07) | 1.19  (0.11) | 1.07***  (0.13) | 2.38  (0.09) | 1.31  (0.10) | 1.07***  (0.13) | 2.29  (0.07) | 1.2  (0.11) | 1.09***  (0.13) | 2.29  (0.07) | 1.2  (0.11) | 1.09***  (0.13) |
| **Iron**  **(mg)** | mean (SE) | 3.25  (0.13) | 3.43  (0.24) | **-**0.18  (0.27) | 3.3  (0.19) | 3.39  (0.21) | **-**0.09  (0.28) | 3.23  (0.13) | 3.44  (0.23) | **-**0.22  (0.27) | 3.23  (0.13) | 3.44  (0.23) | **-**0.22  (0.27) |

^a^ We defined and applied four different carb ratios: per 10g of carb:(a) ≥1g fiber (10:1 carb:fiber), (b) ≥1g fiber and <1g free sugars (10:1:1 carb:fiber:free sugars), (c) ≥1g fiber and <2g free sugars (10:1:2 carb:fiber:free sugars); and (d) ≥1g fiber and, per each 1 g of fiber, <2g free sugars (10:1 carb:fiber, 1:2 fiber:free sugars; or 10:1|1:2).

^b^ All units were expressed as per 100 grams.

^c^ The amount of nutrients contained in each of the food code per 100 grams available in NHANES was calculated using US Department of Agriculture’s Food and Nutrient Database for Dietary Studies.

^d^ Statistical significances for differences (Dif.) is noted as ****P*<0.001, ***P*<0.01 and **P*<0.05.

^e^ Total sugars included both added sugar and natural sugar such as lactose present in milk and fructose present in whole or cut fruit and 100% fruit juice; added sugars included sugars that were added to foods as an ingredient during preparation, processing, or at the table; and free sugars included added sugars (e.g., honey, white sugar, syrups), sugar present in beverages (excluding sugars from dairy), sugar from fruit juice, and sugars from extruded fruit/vegetable products.

# **Table S10. Nutrient composition of available quick breads and bread products meeting or not meeting each of four proposed metrics for assessing carbohydrate quality in the US.**

|  |  | **Meeting or not Meeting each Metric**^s^ | | | | | | | | | | | |
| --- | --- | --- | --- | --- | --- | --- | --- | --- | --- | --- | --- | --- | --- |
| **Composition**^b^ | **Weighted**  **Values**^c^ | **10:1** | | | **10:1:1** | | | **10:1:2** | | | **10:1\|1:2** | | |
|  |  | **Yes** | **No** | **Dif.**^d^ | **Yes** | **No** | **Dif.** ^d^ | **Yes** | **No** | **Dif.** ^d^ | **Yes** | **No** | **Dif.** ^d^ |
|  | # (%) products | 10  (7.0%) | 133  (93.0%) |  | 5  (3.5%) | 138  (96.5%) |  | 9  (6.3%) | 134  (93.7%) |  | 10  (7.0%) | 133  (93.0%) |  |
| **Calories**  **(kJ)** | mean (SE) | 249.9  (20.9) | 284.2  (5.8) | **-**34.3  (21.9) | 240.2  (33.6) | 283.3  (5.7) | **-**43.1  (30.4) | 248.1  (23.3) | 284.1  (5.7) | **-**36  (23) | 249.9  (20.9) | 284.2  (5.8) | **-**34.3  (21.9) |
| **Total fat**  **(g)** | mean (SE) | 9.46  (2.26) | 11.08  (0.49) | **-**1.62  (1.87) | 10.2  (2.93) | 10.99  (0.49) | **-**0.79  (2.61) | 9.55  (2.52) | 11.06  (0.48) | **-**1.51  (1.97) | 9.46  (2.26) | 11.08  (0.49) | **-**1.62  (1.87) |
| **Saturated fat (g)** | mean (SE) | 1.97  (0.40) | 2.87  (0.16) | **-**0.90  (0.61) | 2.34  (0.52) | 2.83  (0.16) | **-**0.48  (0.85) | 2.05  (0.43) | 2.86  (0.16) | **-**0.81  (0.64) | 1.97  (0.40) | 2.87  (0.16) | **-**0.90  (0.61) |
| **Protein**  **(g)** | mean (SE) | 8.1  (0.63) | 6.8  (0.17) | 1.3*  (0.64) | 7.87  (1.13) | 6.86  (0.17) | 1.01  (0.9) | 8.05  (0.7) | 6.82  (0.17) | 1.23  (0.68) | 8.1  (0.63) | 6.8  (0.17) | 1.3*  (0.64) |
| **Total sugar (g)**^d^ | mean (SE) | 5.86  (1.19) | 10.46  (0.66) | **-**4.6  (2.44) | 3.2  (0.8) | 10.39  (0.64) | **-**7.19*  (3.38) | 4.91  (0.8) | 10.49  (0.66) | **-**5.58*  (2.55) | 5.86  (1.19) | 10.46  (0.66) | **-**4.6  (2.44) |
| **Added sugar (g)**^d^ | mean (SE) | 3.58  (1.0) | 7.82  (0.64) | **-**4.24  (2.37) | 1.36  (0.56) | 7.75  (0.62) | **-**6.39  (3.29) | 2.79  (0.69) | 7.84  (0.64) | **-**5.05*  (2.48) | 3.58  (1.0) | 7.82  (0.64) | **-**4.24  (2.37) |
| **Free sugar (g)**^d^ | mean (SE) | 3.58  (1.0) | 7.84  (0.64) | **-**4.26  (2.37) | 1.36  (0.56) | 7.77  (0.62) | **-**6.41  (3.28) | 2.79  (0.69) | 7.86  (0.64) | **-**5.07*  (2.48) | 3.58  (1.0) | 7.84  (0.64) | **-**4.26  (2.37) |
| **Fiber**  **(g)** | mean (SE) | 4.55  (0.52) | 2.01  (0.08) | 2.54***  (0.33) | 3.84  (0.65) | 2.13  (0.10) | 1.71**  (0.52) | 4.39  (0.55) | 2.04  (0.09) | 2.35***  (0.36) | 4.55  (0.52) | 2.01  (0.08) | 2.54***  (0.33) |
| **Sodium**  **(mg)** | mean (SE) | 465  (70.1) | 493.9  (18.1) | **-**28.9  (68.7) | 408.4  (124.6) | 494.9  (17.6) | **-**86.5  (95.2) | 443.1  (74.5) | 495.1  (18) | **-**52  (72.1) | 465  (70.1) | 493.9  (18.1) | **-**28.9  (68.7) |
| **Potassium (mg)** | mean (SE) | 278.1  (37.3) | 173.7  (11.6) | 104.4*  (43.7) | 293.4  (75.3) | 176.9  (11.3) | 116.5  (61.1) | 274.8  (41.5) | 174.7  (11.6) | 100.1*  (46.1) | 278.1  (37.3) | 173.7  (11.6) | 104.4*  (43.7) |
| **Magnesium (mg)** | mean (SE) | 64.6  (8.5) | 26.1  (1.6) | 38.5***  (6.1) | 55  (13.3) | 27.9  (1.7) | 27.1**  (9.4) | 61.7  (8.9) | 26.6  (1.6) | 35***  (6.7) | 64.6  (8.5) | 26.1  (1.6) | 38.5***  (6.1) |
| **Folic acid**  **(mcg)** | mean (SE) | 10.9  (5.4) | 34.8  (1.7) | **-**23.9***  (6.4) | 2.8  (1.8) | 34.2  (1.7) | **-**31.4**  (9) | 8.9  (5.6) | 34.7  (1.7) | **-**25.8***  (6.7) | 10.9  (5.4) | 34.8  (1.7) | **-**23.9***  (6.4) |
| **Thiamine**  **(mg)** | mean (SE) | 0.26  (0.05) | 0.25  (0.01) | 0.01  (0.04) | 0.20  (0.02) | 0.25  (0.01) | **-**0.05  (0.06) | 0.26  (0.05) | 0.25  (0.01) | 0.01  (0.04) | 0.26  (0.05) | 0.25  (0.01) | 0.01  (0.04) |
| **Riboflavin**  **(mg)** | mean (SE) | 0.25  (0.04) | 0.29  (0.01) | **-**0.03  (0.04) | 0.19  (0.02) | 0.29  (0.01) | **-**0.10  (0.06) | 0.24  (0.04) | 0.29  (0.01) | **-**0.05  (0.04) | 0.25  (0.04) | 0.29  (0.01) | **-**0.03  (0.04) |
| **Niacin**  **(mg)** | mean (SE) | 2.73  (0.54) | 2.44  (0.14) | 0.29  (0.52) | 1.92  (0.31) | 2.48  (0.14) | 0.55  (0.73) | 2.68  (0.6) | 2.44  (0.14) | 0.24  (0.55) | 2.73  (0.54) | 2.44  (0.14) | 0.29  (0.52) |
| **Vitamin B6 (mcg)**^d^ | mean (SE) | 0.24  (0.04) | 0.16  (0.02) | 0.08  (0.07) | 0.20  (0.02) | 0.16  (0.02) | 0.03  (0.09) | 0.24  (0.05) | 0.16  (0.02) | 0.08  (0.07) | 0.24  (0.04) | 0.16  (0.02) | 0.08  (0.07) |
| **Vitamin B12 (mcg)**^d^ | mean (SE) | 0.47  (0.23) | 0.48  (0.06) | 0 | 0.24  (0.04) | 0.48  (0.06) | 0.25  (0.32) | 0.49  (0.26) | 0.47  (0.06) | 0.02  (0.24) | 0.47  (0.23) | 0.48  (0.06) | 0 |
| **Vitamin E**  **(mcg)** | mean (SE) | 1.04  (0.22) | 1.05  (0.06) | **-**0.01  (0.21) | 1.17  (0.37) | 1.04  (0.05) | 0.13  (0.29) | 1.03  (0.24) | 1.05  (0.05) | **-**0.02  (0.22) | 1.04  (0.22) | 1.05  (0.06) | **-**0.01  (0.21) |
| **Zinc**  **(mg)** | mean (SE) | 1.32  (0.13) | 0.74  (0.02) | 0.58***  (0.09) | 1.2  (0.23) | 0.77  (0.03) | 0.43**  (0.14) | 1.32  (0.15) | 0.75  (0.02) | 0.57***  (0.1) | 1.32  (0.13) | 0.74  (0.02) | 0.58***  (0.09) |
| **Iron**  **(mg)** | mean (SE) | 2.5  (0.46) | 2.43  (0.12) | 0.06  (0.46) | 1.76  (0.26) | 2.46  (0.12) | **-**0.70  (0.63) | 2.45  (0.51) | 2.44  (0.12) | 0.02  (0.48) | 2.5  (0.46) | 2.43  (0.12) | 0.06  (0.46) |

^a^ We defined and applied four different carb ratios: per 10g of carb:(a) ≥1g fiber (10:1 carb:fiber), (b) ≥1g fiber and <1g free sugars (10:1:1 carb:fiber:free sugars), (c) ≥1g fiber and <2g free sugars (10:1:2 carb:fiber:free sugars); and (d) ≥1g fiber and, per each 1 g of fiber, <2g free sugars (10:1 carb:fiber, 1:2 fiber:free sugars; or 10:1|1:2).

^b^ All units were expressed as per 100 grams.

^c^ The amount of nutrients contained in each of the food code per 100 grams available in NHANES was calculated using US Department of Agriculture’s Food and Nutrient Database for Dietary Studies.

^d^ Statistical significances for differences (Dif.) is noted as ****P*<0.001, ***P*<0.01 and **P*<0.05.

^e^ Total sugars included both added sugar and natural sugar such as lactose present in milk and fructose present in whole or cut fruit and 100% fruit juice; added sugars included sugars that were added to foods as an ingredient during preparation, processing, or at the table; and free sugars included added sugars (e.g., honey, white sugar, syrups), sugar present in beverages (excluding sugars from dairy), sugar from fruit juice, and sugars from extruded fruit/vegetable products.

# **Table S11. Nutrient composition of available cold cereals products meeting or not meeting each of four proposed metrics for assessing carbohydrate quality in the US**

|  |  | **Meeting or not Meeting each Metric**^s^ | | | | | | | | | | | |
| --- | --- | --- | --- | --- | --- | --- | --- | --- | --- | --- | --- | --- | --- |
| **Composition**^b^ | **Weighted**  **Values**^c^ | **10:1** | | | **10:1:1** | | | **10:1:2** | | | **10:1\|1:2** | | |
|  |  | **Yes** | **No** | **Dif.**^d^ | **Yes** | **No** | **Dif.** ^d^ | **Yes** | **No** | **Dif.** ^d^ | **Yes** | **No** | **Dif.** ^d^ |
|  | # (%) products | 87  (43.9%) | 111  (56.1%) |  | 18  (9.1%) | 180  (80.9%) |  | 41  (20.7%) | 157  (79.3%) |  | 68  (34.3%) | 130  (65.7%) |  |
| **Calories**  **(kJ)** | mean (SE) | 358.7  (4.1) | 385.9  (1.3) | **-**27.2***  (4.3) | 350.2  (8.8) | 376.3  (2.1) | **-**26.1***  (9.1) | 354  (5.7) | 379.2  (2.1) | **-**25.1***  (6.1) | 353.7  (4.8) | 384.6  (1.4) | **-**30.9***  (5.0) |
| **Total fat**  **(g)** | mean (SE) | 4.94  (0.40) | 3.94  (0.21) | 1.0*  (0.46) | 4.25  (0.63) | 4.39  (0.23) | **-**0.14  (0.67) | 4.54  (0.42) | 4.34  (0.25) | 0.21  (0.49) | 4.77  (0.44) | 4.18  (0.24) | 0.59  (0.50) |
| **Saturated fat (g)** | mean (SE) | 1.01  (0.12) | 1.06  (0.1) | **-**0.05  (0.16) | 0.73  (0.10) | 1.07  (0.08) | **-**0.33  (0.13) | 0.73  (0.06) | 1.12  (0.10) | **-**0.39*  (0.11) | 0.76  (0.07) | 1.18  (0.11) | **-**0.43**  (0.13) |
| **Protein**  **(g)** | mean (SE) | 9.83  (0.4) | 6.52  (0.2) | 3.31***  (0.45) | 11.87  (0.59) | 7.58  (0.24) | 4.29***  (0.63) | 10.71  (0.53) | 7.26  (0.24) | 3.45***  (0.58) | 10.26  (0.4) | 6.78  (0.24) | 3.49***  (0.47) |
| **Total sugar (g)**^d^ | mean (SE) | 19.12  (1.08) | 30.17  (1.14) | **-**11.05***  (1.57) | 5.75  (1.72) | 27.27  (0.83) | **-**21.53***  (1.91) | 13.17  (1.48) | 28.49  (0.89) | **-**15.31***  (1.72) | 16.56  (1.08) | 29.9  (1.01) | **-**13.34***  (1.48) |
| **Added sugar (g)**^d^ | mean (SE) | 15.12  (1.02) | 28.77  (1.14) | **-**13.65***  (1.58) | 1.63  (0.53) | 24.89  (0.86) | **-**23.26***  (2.74) | 7.78  (0.94) | 26.69  (0.9) | **-**18.9***  (1.83) | 11.94  (0.87) | 28.44  (1.02) | **-**16.5***  (1.54) |
| **Free sugar (g)**^d^ | mean (SE) | 15.12  (1.02) | 28.77  (1.15) | **-**13.65***  (1.54) | 1.63  (0.51) | 24.89  (0.86) | **-**23.26***  (1.01) | 7.78  (0.93) | 26.69  (0.90) | **-**18.9***  (1.29) | 11.94  (0.87) | 28.44  (1.02) | **-**16.5***  (1.34) |
| **Fiber**  **(g)** | mean (SE) | 12.47  (0.67) | 4.37  (0.22) | 8.1***  (0.71) | 12.75  (2.1) | 7.45  (0.41) | 5.3***  (2.09) | 12.37  (1.06) | 6.77  (0.42) | 5.6***  (1.14) | 13.28  (0.83) | 5.14  (0.25) | 8.14***  (0.87) |
| **Sodium**  **(mg)** | mean (SE) | 340.2  (23.3) | 511.9  (17.4) | **-**171.7***  (29.1) | 273.9  (50.5) | 452.7  (15.6) | **-**178.8**  (52.9) | 297.4  (32.1) | 472.8  (16.3) | **-**175.4***  (36.0) | 321.5  (26.6) | 496.6  (16.5) | **-**175.1***  (31.3) |
| **Potassium (mg)** | mean (SE) | 405.2  (18.8) | 192.8  (7.7) | 212.4***  (20.3) | 440.3  (30.3) | 270.7  (12.2) | 169.6***  (32.7) | 440.4  (25.8) | 245.9  (11.5) | 194.5***  (28.3) | 434  (18.9) | 208.8  (9.9) | 225.2***  (21.4) |
| **Magnesium (mg)** | mean (SE) | 103.9  (6.5) | 46.3  (2.4) | 57.6***  (6.9) | 122.8  (8.7) | 66.5  (3.8) | 56.3***  (9.5) | 114.2  (10.0) | 60.5  (3.4) | 53.7***  (10.6) | 111.6  (7.1) | 50.7  (3.0) | 61***  (7.7) |
| **Folic acid**  **(mcg)** | mean (SE) | 393  (42) | 560.2  (33.7) | **-**167.2**  (53.8) | 339  (72.6) | 501.5  (28.6) | **-**162.5*  (78.1) | 340.8  (56) | 524.9  (30.1) | **-**184.1**  (63.3) | 403  (52) | 530.6  (30.3) | **-**127.5*  (60.1) |
| **Thiamine**  **(mg)** | mean (SE) | 1.04  (0.1) | 1.36  (0.05) | **-**0.32**  (0.12) | 0.84  (0.12) | 1.26  (0.06) | **-**0.41*  (0.13) | 0.86  (0.1) | 1.31  (0.06) | **-**0.45**  (0.12) | 1.0  (0.13) | 1.33  (0.05) | **-**0.34**  (0.14) |
| **Riboflavin**  **(mg)** | mean (SE) | 1.04  (0.12) | 1.45  (0.06) | **-**0.41**  (0.14) | 0.41  (0.11) | 1.36  (0.07) | **-**0.95***  (0.13) | 0.71  (0.11) | 1.42  (0.07) | **-**0.71***  (0.14) | 0.97  (0.15) | 1.43  (0.06) | **-**0.46**  (0.16) |
| **Niacin**  **(mg)** | mean (SE) | 13.22  (1.4) | 17.82  (0.72) | **-**4.6**  (1.58) | 10.69  (1.59) | 16.31  (0.81) | **-**5.62*  (1.78) | 10.45  (1.24) | 17.2  (0.86) | **-**6.75***  (1.51) | 12.74  (1.74) | 17.4  (0.66) | **-**4.66**  (1.86) |
| **Vitamin B6 (mcg)**^d^ | mean (SE) | 1.64  (0.21) | 1.98  (0.09) | **-**0.34  (0.23) | 1.37  (0.28) | 1.87  (0.11) | **-**0.50  (0.30) | 1.59  (0.33) | 1.89  (0.1) | **-**0.30  (0.34) | 1.67  (0.26) | 1.91  (0.08) | **-**0.24  (0.27) |
| **Vitamin B12 (mcg)**^d^ | mean (SE) | 4.15  (0.56) | 5.31  (0.31) | **-**1.16  (0.64) | 3.34  (0.92) | 4.94  (0.32) | **-**1.61  (0.98) | 3.69  (0.73) | 5.09  (0.33) | **-**1.39  (0.80) | 4.1  (0.70) | 5.16  (0.28) | **-**1.06  (0.75) |
| **Vitamin E**  **(mcg)** | mean (SE) | 4.52  (1.25) | 2.9  (0.71) | 1.61  (1.44) | 1.65  (0.69) | 3.81  (0.74) | **-**2.16*  (1.01) | 2.74  (1.48) | 3.84  (0.77) | **-**1.1  (1.66) | 5.37  (1.56) | 2.7  (0.62) | 2.67  (1.68) |
| **Zinc**  **(mg)** | mean (SE) | 7.69  (1.1) | 8.64  (0.68) | **-**0.96  (1.29) | 7.01  (1.44) | 8.34  (0.66) | **-**1.33  (1.59) | 6.49  (1.02) | 8.67  (0.73) | **-**2.18  (1.25) | 8.32  (1.35) | 8.17  (0.62) | 0.15  (1.49) |
| **Iron**  **(mg)** | mean (SE) | 17.39  (1.52) | 18.18  (0.99) | **-**0.79  (1.81) | 16.13  (3.0) | 18  (0.91) | **-**1.87  (3.16) | 14.99  (1.85) | 18.57  (0.97) | **-**3.58  (2.09) | 17.38  (1.84) | 18.07  (0.91) | **-**0.69  (2.05) |

^a^ We defined and applied four different carb ratios: per 10g of carb:(a) ≥1g fiber (10:1 carb:fiber), (b) ≥1g fiber and <1g free sugars (10:1:1 carb:fiber:free sugars), (c) ≥1g fiber and <2g free sugars (10:1:2 carb:fiber:free sugars); and (d) ≥1g fiber and, per each 1 g of fiber, <2g free sugars (10:1 carb:fiber, 1:2 fiber:free sugars; or 10:1|1:2).

^b^ All units were expressed as per 100 grams.

^c^ The amount of nutrients contained in each of the food code per 100 grams available in NHANES was calculated using US Department of Agriculture’s Food and Nutrient Database for Dietary Studies.

^d^ Statistical significances for differences (Dif.) is noted as ****P*<0.001, ***P*<0.01 and **P*<0.05.

^e^ Total sugars included both added sugar and natural sugar such as lactose present in milk and fructose present in whole or cut fruit and 100% fruit juice; added sugars included sugars that were added to foods as an ingredient during preparation, processing, or at the table; and free sugars included added sugars (e.g., honey, white sugar, syrups), sugar present in beverages (excluding sugars from dairy), sugar from fruit juice, and sugars from extruded fruit/vegetable products.

# **Table S12. Nutrient composition of available savory snacks products meeting or not meeting each of four proposed metrics for assessing carbohydrate quality in the US**

|  |  | **Meeting or not Meeting each Metric**^s^ | | | | | | | | | | | |
| --- | --- | --- | --- | --- | --- | --- | --- | --- | --- | --- | --- | --- | --- |
| **Composition**^b^ | **Weighted**  **Values**^c^ | **10:1** | | | **10:1:1** | | | **10:1:2** | | | **10:1\|1:2** | | |
|  |  | **Yes** | **No** | **Dif.**^d^ | **Yes** | **No** | **Dif.** ^d^ | **Yes** | **No** | **Dif.** ^d^ | **Yes** | **No** | **Dif.** ^d^ |
|  | # (%) products | 46  (26.0%) | 131  (74%) |  | 44  (24.9%) | 133  (75.1%) |  | 44  (24.9%) | 133  (75.1%) |  | 45  (25.4%) | 132  (75.6%) |  |
| **Calories**  **(kJ)** | mean (SE) | 488.7  (9.4) | 444  (6.3) | 44.7***  (11.3) | 491.9  (9.6) | 443.6  (6.2) | 48.2***  (11.4) | 491.9  (9.5) | 443.6  (6.2) | 48.2***  (11.4) | 490.6  (9.4) | 443.7  (6.3) | 46.8***  (11.3) |
| **Total fat**  **(g)** | mean (SE) | 24.47  (1.68) | 17.24  (1.04) | 7.23***  (1.98) | 24.99  (1.71) | 17.17  (1.03) | 7.82***  (2.00) | 24.99  (1.71) | 17.17  (1.03) | 7.82***  (2.00) | 24.73  (1.70) | 17.2  (1.04) | 7.52***  (1.99) |
| **Saturated fat (g)** | mean (SE) | 6.45  (1.05) | 3.48  (0.22) | 2.97***  (1.08) | 6.61  (1.09) | 3.47  (0.22) | 3.14***  (1.12) | 6.61  (1.09) | 3.47  (0.22) | 3.14***  (1.12) | 6.5  (1.07) | 3.48  (0.22) | 3.02***  (1.10) |
| **Protein**  **(g)** | mean (SE) | 9.19  (0.40) | 7.45  (0.24) | 1.74***  (0.47) | 9.28  (0.41) | 7.44  (0.24) | 1.84***  (0.47) | 9.28  (0.41) | 7.44  (0.24) | 1.84***  (0.47) | 9.27  (0.40) | 7.43  (0.24) | 1.84***  (0.47) |
| **Total sugar (g)**^d^ | mean (SE) | 3.13  (1.12) | 5.89  (1.03) | **-**2.76  (1.52) | 1.73  (0.36) | 6.31  (1.07) | **-**4.59*  (1.13) | 1.73  (0.36) | 6.31  (1.07) | **-**4.59*  (1.13) | 2.14  (0.54) | 6.21  (1.07) | **-**4.07*  (1.20) |
| **Added sugar (g)**^d^ | mean (SE) | 2.0  (1.10) | 4.06  (0.99) | **-**2.06  (1.48) | 0.59  (0.28) | 4.5  (1.03) | **-**3.91*  (1.07) | 0.59  (0.28) | 4.5  (1.03) | **-**3.91*  (1.07) | 1.01  (0.51) | 4.38  (1.03) | **-**3.37  (1.15) |
| **Free sugar (g)**^d^ | mean (SE) | 2.0  (1.10) | 4.06  (0.99) | **-**2.06  (1.48) | 0.59  (0.28) | 4.5  (1.03) | **-**3.91*  (1.07) | 0.59  (0.28) | 4.5  (1.03) | **-**3.91*  (1.07) | 1.01  (0.51) | 4.38  (1.03) | **-**3.37  (1.15) |
| **Fiber**  **(g)** | mean (SE) | 10.35  (0.31) | 3.69  (0.13) | 6.66***  (0.34) | 10.41  (0.33) | 3.77  (0.14) | 6.64***  (0.35) | 10.41  (0.33) | 3.77  (0.14) | 6.64***  (0.35) | 10.4  (0.32) | 3.72  (0.13) | 6.68***  (0.34) |
| **Sodium**  **(mg)** | mean (SE) | 553.2  (35.4) | 664.1  (36.1) | **-**110.9*  (50.6) | 555.7  (37.0) | 661.6  (35.6) | **-**105.9*  (51.3) | 555.7  (37.0) | 661.6  (35.6) | **-**105.9*  (51.3) | 554.5  (36.2) | 662.8  (35.8) | **-**108.3*  (50.9) |
| **Potassium (mg)** | mean (SE) | 339.3  (32.2) | 440.9  (40.4) | **-**101.6  (51.7) | 343.9  (33.5) | 437.8  (39.8) | **-**93.9  (52.1) | 343.9  (33.5) | 437.8  (39.8) | **-**93.9  (52.1) | 341.3  (32.9) | 439.4  (40.1) | **-**98.1  (51.9) |
| **Magnesium (mg)** | mean (SE) | 103.5  (3.5) | 50.5  (2.6) | 53***  (4.3) | 104  (3.6) | 51.1  (2.6) | 52.9***  (4.4) | 104  (3.6) | 51.1  (2.6) | 52.9***  (4.4) | 104  (3.5) | 50.7  (2.6) | 53.2***  (4.4) |
| **Folic acid**  **(mcg)** | mean (SE) | 0.70  (0.3) | 16.5  (3.5) | **-**15.8***  (3.5) | 0.73  (0.4) | 16.3  (3.5) | **-**15.6***  (3.49) | 0.73  (0.35) | 16.3  (3.5) | **-**15.6***  (3.49) | 0.71  (0.34) | 16.4  (3.5) | **-**15.7***  (3.51) |
| **Thiamine**  **(mg)** | mean (SE) | 0.14  (0.02) | 0.25  (0.01) | **-**0.11***  (0.02) | 0.14  (0.02) | 0.25  (0.01) | **-**0.11***  (0.02) | 0.14  (0.02) | 0.25  (0.01) | **-**0.11***  (0.02) | 0.14  (0.02) | 0.25  (0.01) | **-**0.11***  (0.02) |
| **Riboflavin**  **(mg)** | mean (SE) | 0.09  (0.01) | 0.20  (0.01) | **-**0.11***  (0.01) | 0.09  (0.01) | 0.20  (0.01) | **-**0.11***  (0.01) | 0.09  (0.01) | 0.20  (0.01) | **-**0.11***  (0.01) | 0.09  (0.01) | 0.20  (0.01) | **-**0.11***  (0.01) |
| **Niacin**  **(mg)** | mean (SE) | 1.83  (0.09) | 3.4  (0.17) | **-**1.58***  (0.19) | 1.82  (0.09) | 3.38  (0.17) | **-**1.56***  (0.19) | 1.82  (0.09) | 3.38  (0.17) | **-**1.56***  (0.19) | 1.82  (0.09) | 3.39  (0.17) | **-**1.57***  (0.19) |
| **Vitamin B6 (mcg)**^d^ | mean (SE) | 0.22  (0.02) | 0.23  (0.02) | **-**0.01  (0.02) | 0.23  (0.02) | 0.23  (0.02) | 0 | 0.23  (0.02) | 0.23  (0.02) | 0 | 0.22  (0.02) | 0.23  (0.02) | 0 |
| **Vitamin B12 (mcg)**^d^ | mean (SE) | 0.04  (0.02) | 0.04  (0.01) | 0.01  (0.02) | 0.04  (0.02) | 0.04  (0.01) | 0 | 0.04  (0.02) | 0.04  (0.01) | 0 | 0.04  (0.02) | 0.04  (0.01) | 0.01  (0.02) |
| **Vitamin E**  **(mcg)** | mean (SE) | 3.24  (0.36) | 3.53  (0.30) | **-**0.28  (0.47) | 3.33  (0.37) | 3.49  (0.30) | **-**0.16  (0.47) | 3.33  (0.37) | 3.49  (0.30) | **-**0.16  (0.47) | 3.29  (0.36) | 3.51  (0.30) | **-**0.22  (0.47) |
| **Zinc**  **(mg)** | mean (SE) | 2.34  (0.11) | 1.02  (0.04) | 1.32***  (0.12) | 2.37  (0.12) | 1.03  (0.04) | 1.34***  (0.12) | 2.37  (0.12) | 1.03  (0.04) | 1.34***  (0.12) | 2.36  (0.11) | 1.02  (0.04) | 1.34***  (0.12) |
| **Iron**  **(mg)** | mean (SE) | 2.29  (0.07) | 2.22  (0.12) | 0.08  (0.14) | 2.28  (0.07) | 2.22  (0.11) | 0.06  (0.14) | 2.28  (0.07) | 2.22  (0.11) | 0.06  (0.14) | 2.28  (0.07) | 2.22  (0.12) | 0.06  (0.14) |

^a^ We defined and applied four different carb ratios: per 10g of carb:(a) ≥1g fiber (10:1 carb:fiber), (b) ≥1g fiber and <1g free sugars (10:1:1 carb:fiber:free sugars), (c) ≥1g fiber and <2g free sugars (10:1:2 carb:fiber:free sugars); and (d) ≥1g fiber and, per each 1 g of fiber, <2g free sugars (10:1 carb:fiber, 1:2 fiber:free sugars; or 10:1|1:2).

^b^ All units were expressed as per 100 grams.

^c^ The amount of nutrients contained in each of the food code per 100 grams available in NHANES was calculated using US Department of Agriculture’s Food and Nutrient Database for Dietary Studies.

^d^ Statistical significances for differences (Dif.) is noted as ****P*<0.001, ***P*<0.01 and **P*<0.05.

^e^ Total sugars included both added sugar and natural sugar such as lactose present in milk and fructose present in whole or cut fruit and 100% fruit juice; added sugars included sugars that were added to foods as an ingredient during preparation, processing, or at the table; and free sugars included added sugars (e.g., honey, white sugar, syrups), sugar present in beverages (excluding sugars from dairy), sugar from fruit juice, and sugars from extruded fruit/vegetable products.

# **Table S13. Nutrient composition of available sweet bakery products meeting or not meeting each of four proposed metrics for assessing carbohydrate quality in the US**

|  |  | **Meeting or not Meeting each Metric**^s^ | | | | | | | | | | | |
| --- | --- | --- | --- | --- | --- | --- | --- | --- | --- | --- | --- | --- | --- |
| **Composition**^b^ | **Weighted**  **Values**^c^ | **10:1** | | | **10:1:1** | | | **10:1:2** | | | **10:1\|1:2** | | |
|  |  | **Yes** | **No** | **Dif.**^d^ | **Yes** | **No** | **Dif.** ^d^ | **Yes** | **No** | **Dif.** ^d^ | **Yes** | **No** | **Dif.** ^d^ |
|  | # (%) products | 10  (2.6%) | 376  (97.4%) |  | 3  (0.78%) | 383  (99.2%) |  | 3  (0.78%) | 383  (99.2%) |  | 5  (1.29%) | 381  (98.7%) |  |
| **Calories**  **(kJ)** | mean (SE) | 366.6  (28.4) | 359.7  (4.6) | 6.9  (28.8) | 450.7  (32.9) | 359.1  (4.6) | 91.5**  (33.2) | 450.7  (32.9) | 359.1  (4.6) | 91.5**  (33.2) | 425.8  (24.9) | 359  (4.6) | 66.8**  (25.3) |
| **Total fat**  **(g)** | mean (SE) | 12.72  (1.72) | 15.95  (0.36) | **-**3.24  (1.76) | 17.57  (4.52) | 15.86  (0.36) | 1.71  (4.54) | 17.57  (4.52) | 15.86  (0.36) | 1.71  (4.54) | 14.78  (3.12) | 15.89  (0.36) | **-**1.11  (3.14) |
| **Saturated fat (g)** | mean (SE) | 3.99  (0.36) | 5.11  (0.19) | **-**1.12**  (0.41) | 4.51  (0.55) | 5.08  (0.19) | **-**0.57  (0.49) | 4.51  (0.45) | 5.08  (0.19) | **-**0.57  (0.49) | 4.27  (0.31) | 5.09  (0.19) | **-**0.82*  (0.36) |
| **Protein**  **(g)** | mean (SE) | 3.26  (0.20) | 4.72  (0.10) | **-**1.46***  (0.22) | 3.66  (0.04) | 4.69  (0.10) | **-**1.02***  (0.10) | 3.66  (0.05) | 4.69  (0.10) | **-**1.02***  (0.10) | 3.74  (0.07) | 4.69  (0.10) | **-**0.95***  (0.12) |
| **Total sugar (g)**^d^ | mean (SE) | 27.1  (3.66) | 25.68  (0.60) | 1.42  (3.71) | 21.04  (8.66) | 25.75  (0.59) | **-**4.71  (8.68) | 21.04  (8.66) | 25.75  (0.59) | **-**4.71  (8.68) | 25  (5.64) | 25.72  (0.59) | **-**0.73  (5.67) |
| **Added sugar (g)**^d^ | mean (SE) | 19.59  (4.78) | 22.46  (0.62) | **-**2.87  (3.91) | 0 | 22.56  (0.62) | **-**22.56**  (6.99) | 0 | 22.56  (0.62) | **-**22.56**  (6.99) | 11.77  (7.24) | 22.53  (0.62) | **-**10.76*  (5.47) |
| **Free sugar (g)**^d^ | mean (SE) | 19.6  (4.78) | 22.58  (0.63) | **-**2.98  (4.82) | 0 | 22.68  (0.62) | **-**22.68**  (0.62) | 0 | 22.68  (0.62) | **-**22.68**  (0.62) | 11.77  (6.49) | 22.64  (0.63) | **-**10.87*  (6.52) |
| **Fiber**  **(g)** | mean (SE) | 11.86  (1.46) | 1.76  (0.05) | 10.1***  (1.47) | 14.7  (0.16) | 1.92  (0.09) | 12.78***  (0.19) | 14.7  (0.16) | 1.92  (0.09) | 12.78***  (0.19) | 15.02  (0.26) | 1.85  (0.07) | 13.17***  (0.27) |
| **Sodium**  **(mg)** | mean (SE) | 305.6  (50.4) | 302.3  (6.7) | 3.3  (50.9) | 290  (97.9) | 302.5  (6.6) | **-**12.5  (98.2) | 290  (97.9) | 302.5  (6.6) | **-**12.5  (98.2) | 349.6  (75.8) | 301.8  (6.6) | 47.8  (76.1) |
| **Potassium (mg)** | mean (SE) | 133.2  (21) | 130.6  (3.2) | 2.6  (21.2) | 107.7  (35.1) | 130.8  (3.2) | **-**23.2  (35.3) | 107.7  (35.1) | 130.8  (3.2) | **-**23.2  (35.3) | 134  (38.3) | 130.6  (3.2) | 3.4  (38.4) |
| **Magnesium (mg)** | mean (SE) | 21.8  (3.4) | 20.2  (0.71) | 1.6  (3.51) | 17.3  (6.89) | 20.3  (0.70) | **-**2.9  (6.92) | 17.3  (6.89) | 20.3  (0.70) | **-**2.9  (6.92) | 19  (5.2) | 20.2  (0.70) | **-**1.2  (5.2) |
| **Folic acid**  **(mcg)** | mean (SE) | 37.9  (4.95) | 32  (1.34) | 5.9  (5.13) | 48.7  (4.59) | 32.1  (1.32) | 16.6***  (4.78) | 48.7  (4.59) | 32.1  (1.32) | 16.6***  (4.78) | 47  (4.6) | 32  (1.3) | 15.0  (4.8) |
| **Thiamine**  **(mg)** | mean (SE) | 0.22  (0.02) | 0.20  (0.01) | 0.02  (0.02) | 0.27  (0.01) | 0.20  (0.01) | 0.08***  (0.02) | 0.27  (0.01) | 0.20  (0.01) | 0.08***  (0.02) | 0.27  (0.01) | 0.20  (0.01) | 0.08***  (0.01) |
| **Riboflavin**  **(mg)** | mean (SE) | 0.24  (0.03) | 0.20  (0.01) | 0.05  (0.03) | 0.25  (0.04) | 0.20  (0.01) | 0.05  (0.04) | 0.25  (0.04) | 0.20  (0.01) | 0.05  (0.04) | 0.29  (0.04) | 0.20  (0.01) | 0.10*  (0.04) |
| **Niacin**  **(mg)** | mean (SE) | 1.67  (0.15) | 1.79  (0.06) | **-**0.12  (0.16) | 2.03  (0.10) | 1.78  (0.06) | 0.25  (0.12) | 2.03  (0.10) | 1.78  (0.06) | 0.25  (0.12) | 1.97  (0.12) | 1.78  (0.06) | 0.19  (0.13) |
| **Vitamin B6 (mcg)**^d^ | mean (SE) | 0.04  (0) | 0.07  (0.01) | **-**0.03***  (0.01) | 0.03  (0.01) | 0.07  (0.01) | **-**0.03**  (0.01) | 0.03  (0.01) | 0.07  (0.01) | **-**0.03***  (0.01) | 0.04  (0.01) | 0.07  (0.01) | **-**0.03***  (0.01) |
| **Vitamin B12 (mcg)**^d^ | mean (SE) | 0.10  (0.03) | 0.09  (0.01) | 0  (0.03) | 0.13  (0.06) | 0.09  (0.01) | 0.04  (0.06) | 0.13  (0.06) | 0.09  (0.01) | 0.04  (0.06) | 0.14  (0.04) | 0.09  (0.01) | 0.05  (0.04) |
| **Vitamin E**  **(mcg)** | mean (SE) | 1.17  (0.21) | 1.2  (0.05) | **-**0.03  (0.22) | 1.76  (0.54) | 1.2  (0.05) | 0.56  (0.54) | 1.76  (0.54) | 1.2  (0.05) | 0.56  (0.54) | 1.49  (0.36) | 1.2  (0.05) | 0.29  (0.36) |
| **Zinc**  **(mg)** | mean (SE) | 0.47  (0.06) | 0.53  (0.01) | **-**0.05  (0.06) | 0.47  (0.13) | 0.53  (0.01) | **-**0.05  (0.13) | 0.47  (0.13) | 0.53  (0.01) | **-**0.05  (0.13) | 0.50  (0.08) | 0.53  (0.01) | **-**0.02  (0.08) |
| **Iron**  **(mg)** | mean (SE) | 1.92  (0.35) | 1.99  (0.07) | **-**0.06  (0.36) | 2.21  (0.74) | 1.98  (0.07) | 0.23  (0.74) | 2.21  (0.74) | 1.98  (0.07) | 0.23  (0.74) | 1.89  (0.48) | 1.98  (0.07) | **-**0.10  (0.48) |

^a^ We defined and applied four different carb ratios: per 10g of carb:(a) ≥1g fiber (10:1 carb:fiber), (b) ≥1g fiber and <1g free sugars (10:1:1 carb:fiber:free sugars), (c) ≥1g fiber and <2g free sugars (10:1:2 carb:fiber:free sugars); and (d) ≥1g fiber and, per each 1 g of fiber, <2g free sugars (10:1 carb:fiber, 1:2 fiber:free sugars; or 10:1|1:2).

^b^ All units were expressed as per 100 grams.

^c^ The amount of nutrients contained in each of the food code per 100 grams available in NHANES was calculated using US Department of Agriculture’s Food and Nutrient Database for Dietary Studies.

^d^ Statistical significances for differences (Dif.) is noted as ****P*<0.001, ***P*<0.01 and **P*<0.05.

^e^ Total sugars included both added sugar and natural sugar such as lactose present in milk and fructose present in whole or cut fruit and 100% fruit juice; added sugars included sugars that were added to foods as an ingredient during preparation, processing, or at the table; and free sugars included added sugars (e.g., honey, white sugar, syrups), sugar present in beverages (excluding sugars from dairy), sugar from fruit juice, and sugars from extruded fruit/vegetable products.

# **Table S14. Nutrient composition of mixed dishes products meeting or not meeting each of four proposed metrics for assessing carbohydrate quality in the US**

|  |  | **Meeting or not Meeting each Metric**^s^ | | | | | | | | | | | |
| --- | --- | --- | --- | --- | --- | --- | --- | --- | --- | --- | --- | --- | --- |
| **Composition**^b^ | **Weighted**  **Values**^c^ | **10:1** | | | **10:1:1** | | | **10:1:2** | | | **10:1\|1:2** | | |
|  |  | **Yes** | **No** | **Dif.**^d^ | **Yes** | **No** | **Dif.** ^d^ | **Yes** | **No** | **Dif.** ^d^ | **Yes** | **No** | **Dif.** ^d^ |
|  | # (%) products | 143  (23.1%) | 476  (76.9%) |  | 135  (21.8%) | 484  (78.2%) |  | 141  (22.8%) | 478  (77.2%) |  | 143  (23.1%) | 476  (76.9%) |  |
| **Calories**  **(kJ)** | mean (SE) | 156.2  (4.5) | 164.4  (3.3) | **-**8.2  (5.5) | 151.3  (4.3) | 165.6  (3.3) | **-**14.4**  (5.4) | 153.9  (4.3) | 165  (3.3) | **-**11.1*  (5.4) | 156.2  (4.5) | 164.4  (3.3) | **-**8.2  (5.5) |
| **Total fat**  **(g)** | mean (SE) | 6.47  (0.36) | 5.91  (0.24) | 0.56  (0.44) | 6.35  (0.38) | 5.95  (0.24) | 0.40  (0.45) | 6.4  (0.37) | 5.94  (0.24) | 0.46  (0.44) | 6.47  (0.37) | 5.91  (0.24) | 0.56  (0.44) |
| **Saturated fat (g)** | mean (SE) | 2.12  (0.16) | 1.89  (0.10) | 0.22  (0.19) | 2.1  (0.17) | 1.9  (0.10) | 0.20  (0.19) | 2.12  (0.16) | 1.89  (0.10) | 0.22  (0.19) | 2.12  (0.16) | 1.89  (0.10) | 0.22  (0.19) |
| **Protein**  **(g)** | mean (SE) | 6.81  (0.27) | 6.34  (0.17) | 0.47  (0.32) | 6.55  (0.26) | 6.42  (0.17) | 0.12  (0.31) | 6.71  (0.26) | 6.37  (0.17) | 0.34  (0.31) | 6.81  (0.27) | 6.34  (0.17) | 0.47  (0.32) |
| **Total sugar (g)**^d^ | mean (SE) | 2.19  (0.14) | 2.47  (0.16) | **-**0.28  (0.22) | 1.89  (0.09) | 2.55  (0.16) | **-**0.66***  (0.19) | 2.07  (0.12) | 2.5  (0.16) | **-**0.43*  (0.20) | 2.19  (0.14) | 2.47  (0.16) | **-**0.28  (0.22) |
| **Added sugar (g)**^d^ | mean (SE) | 0.62  (0.11) | 1.17  (0.16) | **-**0.56**  (0.19) | 0.36  (0.05) | 1.23  (0.16) | **-**0.88**  (0.17) | 0.51  (0.08) | 1.2  (0.16) | **-**0.69***  (0.18) | 0.63  (0.11) | 1.19  (0.16) | **-**0.56**  (0.19) |
| **Free sugar (g)**^d^ | mean (SE) | 0.63  (0.11) | 1.19  (0.16) | **-**0.56**  (0.19) | 0.37  (0.05) | 1.25  (0.16) | **-**0.88**  (0.17) | 0.52  (0.08) | 1.22  (0.16) | **-**0.70*  (0.18) | 0.63  (0.11) | 1.19  (0.16) | **-**0.56**  (0.19) |
| **Fiber**  **(g)** | mean (SE) | 2.59  (0.08) | 1.26  (0.03) | 1.33***  (0.09) | 2.53  (0.08) | 1.3  (0.03) | 1.23***  (0.09) | 2.56  (0.08) | 1.28  (0.03) | 1.29***  (0.08) | 2.59  (0.08) | 1.26  (0.03) | 1.33***  (0.09) |
| **Sodium**  **(mg)** | mean (SE) | 352.1  (13) | 358.7  (8.2) | **-**6.6  (15.4) | 346.7  (13.4) | 360.1  (8.1) | **-**13.4  (15.6) | 351.4  (13.2) | 358.9  (8.2) | **-**7.5  (15.5) | 352.1  (13.0) | 358.7  (8.2) | **-**6.6  (15.4) |
| **Potassium (mg)** | mean (SE) | 187.6  (5.4) | 131.6  (2.8) | 56.1***  (6.1) | 181.9  (5.0) | 134.1  (2.9) | 47.8***  (5.8) | 186  (5.3) | 132.3  (2.8) | 53.7***  (6.0) | 187.6  (5.4) | 131.6  (2.8) | 56.1***  (6.1) |
| **Magnesium (mg)** | mean (SE) | 31.2  (0.94) | 18.8  (0.44) | 12.4***  (1.04) | 30  (0.81) | 19.3  (0.49) | 10.7***  (0.94) | 30.5  (0.82) | 19  (0.47) | 11.5***  (0.95) | 31.2  (0.94) | 18.8  (0.44) | 12.4***  (1.04) |
| **Folic acid**  **(mcg)** | mean (SE) | 8.6  (1.1) | 24.9  (0.8) | **-**16.3***  (1.34) | 8.1  (1.1) | 24.8  (0.8) | **-**16.7***  (1.3) | 8.6  (1.1) | 24.9  (0.8) | **-**16.2***  (1.4) | 8.6  (1.1) | 24.9  (0.8) | **-**16.3***  (1.3) |
| **Thiamine**  **(mg)** | mean (SE) | 0.13  (0.01) | 0.17  (0) | **-**0.04***  (0.01) | 0.13  (0.01) | 0.18  (0) | **-**0.05***  (0.01) | 0.13  (0.01) | 0.17  (0) | **-**0.05***  (0.01) | 0.13  (0.01) | 0.17  (0) | **-**0.04***  (0.01) |
| **Riboflavin**  **(mg)** | mean (SE) | 0.12  (0.01) | 0.12  (0) | 0.01  (0.01) | 0.11  (0.01) | 0.12  (0) | **-**0.01  (0.01) | 0.12  (0.01) | 0.12  (0) | 0.01  (0.01) | 0.12  (0.01) | 0.12  (0) | 0.01  (0.01) |
| **Niacin**  **(mg)** | mean (SE) | 2.28  (0.09) | 2.08  (0.06) | 0.21  (0.11) | 2.2  (0.08) | 2.1  (0.06) | 0.1  (0.10) | 2.22  (0.08) | 2.1  (0.06) | 0.13  (0.10) | 2.28  (0.09) | 2.08  (0.06) | 0.21  (0.11) |
| **Vitamin B6 (mcg)**^d^ | mean (SE) | 0.12  (0) | 0.1  (0) | 0.02***  (0) | 0.12  (0) | 0.1  (0) | 0.02**  (0) | 0.12  (0) | 0.10  (0) | 0.02***  (0) | 0.12  (0) | 0.1  (0) | 0.02***  (0) |
| **Vitamin B12 (mcg)**^d^ | mean (SE) | 0.27  (0.03) | 0.26  (0.02) | 0.01  (0.04) | 0.24  (0.02) | 0.27  (0.02) | **-**0.03  (0.03) | 0.27  (0.03) | 0.26  (0.02) | 0.01  (0.04) | 0.27  (0.03) | 0.26  (0.02) | 0.01  (0.04) |
| **Vitamin E**  **(mcg)** | mean (SE) | 0.91  (0.06) | 0.62  (0.02) | 0.29***  (0.06) | 0.89  (0.06) | 0.63  (0.02) | 0.26***  (0.06) | 0.88  (0.06) | 0.63  (0.02) | 0.25***  (0.06) | 0.91  (0.06) | 0.62  (0.02) | 0.29***  (0.06) |
| **Zinc**  **(mg)** | mean (SE) | 1.04  (0.04) | 0.80  (0.03) | 0.24***  (0.05) | 1.01  (0.04) | 0.81  (0.03) | 0.19**  (0.05) | 1.04  (0.04) | 0.80  (0.03) | 0.23***  (0.05) | 1.04  (0.04) | 0.80  (0.03) | 0.24***  (0.05) |
| **Iron**  **(mg)** | mean (SE) | 1.36  (0.04) | 1.21  (0.03) | 0.15**  (0.05) | 1.33  (0.04) | 1.22  (0.03) | 0.11*  (0.05) | 1.34  (0.04) | 1.21  (0.03) | 0.13*  (0.05) | 1.36  (0.04) | 1.21  (0.03) | 0.15**  (0.05) |

^a^ We defined and applied four different carb ratios: per 10g of carb:(a) ≥1g fiber (10:1 carb:fiber), (b) ≥1g fiber and <1g free sugars (10:1:1 carb:fiber:free sugars), (c) ≥1g fiber and <2g free sugars (10:1:2 carb:fiber:free sugars); and (d) ≥1g fiber and, per each 1 g of fiber, <2g free sugars (10:1 carb:fiber, 1:2 fiber:free sugars; or 10:1|1:2).

^b^ All units were expressed as per 100 grams.

^c^ The amount of nutrients contained in each of the food code per 100 grams available in NHANES was calculated using US Department of Agriculture’s Food and Nutrient Database for Dietary Studies.

^d^ Statistical significances for differences (Dif.) is noted as ****P*<0.001, ***P*<0.01 and **P*<0.05.

^e^ Total sugars included both added sugar and natural sugar such as lactose present in milk and fructose present in whole or cut fruit and 100% fruit juice; added sugars included sugars that were added to foods as an ingredient during preparation, processing, or at the table; and free sugars included added sugars (e.g., honey, white sugar, syrups), sugar present in beverages (excluding sugars from dairy), sugar from fruit juice, and sugars from extruded fruit/vegetable products.

# **Table S15. Comparisons of available and consumed carbohydrate-rich projects meeting each of the three nutrient profiling systems in the US**

| **Food Categories^a^** | **Analysis**^b^ | **Products** | |  | **% Meeting Nutrient Profiling Systems^d^** | | |
| --- | --- | --- | --- | --- | --- | --- | --- |
|  |  | **#** | **Frequency^c^**  **(million)** |  | **Ofcom**  **(Healthy)** | **FSANZ**  **(Health claim)** | **HSR**  **(Green or nutritious)** |
| **Sweet bakery products** | *Available* | 386 | n/a |  | 1.0 | 2.3 | 6.5 |
|  | *Overall* | 313 | 538.1 |  | 1.6 | 1.1 | 1.3 |
|  | *Children* | 226 | 141.7 |  | 1.1 | 0.6 | 0.5 |
|  | *Adults* | 303 | 396.4 |  | 1.8 | 1.2 | 1.6 |
| **Bread, rolls, tortillas** | *Available* | 206 | n/a |  | 74.8 | 79.1 | 78.2 |
|  | *Overall* | 168 | 826.7 |  | 89.7 | 91.1 | 91.7 |
|  | *Children* | 134 | 170.1 |  | 88.6 | 89.0 | 89.2 |
|  | *Adults* | 162 | 656.6 |  | 90.0 | 91.6 | 92.4 |
| **Cold cereals** | *Available* | 198 | n/a |  | 19.2 | 32.8 | 38.9 |
|  | *Overall* | 174 | 295.6 |  | 17.7 | 23.3 | 31.3 |
|  | *Children* | 144 | 105.1 |  | 11.5 | 14.2 | 16.5 |
|  | *Adults* | 165 | 190.5 |  | 21.1 | 28.3 | 39.4 |
| **Cooked cereals** | *Available* | 197 | n/a |  | 87.8 | 94.9 | 88.3 |
|  | *Overall* | 126 | 85.7 |  | 96.2 | 98.0 | 96.8 |
|  | *Children* | 80 | 12.3 |  | 97.9 | 98.9 | 97.4 |
|  | *Adults* | 117 | 73.4 |  | 96.0 | 97.8 | 96.7 |
| **Savory snacks** | *Available* | 177 | n/a |  | 23.2 | 39.0 | 39.0 |
|  | *Overall* | 150 | 447.7 |  | 23.0 | 53.4 | 53.0 |
|  | *Children* | 136 | 138.8 |  | 20.3 | 47.9 | 47.3 |
|  | *Adults* | 144 | 308.9 |  | 24.3 | 55.9 | 55.5 |
| **Quick breads and bread products** | *Available* | 143 | n/a |  | 18.2 | 23.8 | 16.1 |
|  | *Overall* | 114 | 166.5 |  | 7.3 | 5.5 | 4.3 |
|  | *Children* | 95 | 57.9 |  | 8.7 | 5.4 | 4.6 |
|  | *Adults* | 102 | 108.7 |  | 6.5 | 5.5 | 4.2 |
| **Cooked grains** | *Available* | 82 | n/a |  | 90.2 | 92.7 | 86.6 |
|  | *Overall* | 73 | 189.3 |  | 96.4 | 99.3 | 94.7 |
|  | *Children* | 53 | 40.1 |  | 96.4 | 99.5 | 95.0 |
|  | *Adults* | 70 | 149.2 |  | 96.4 | 99.3 | 94.7 |
| **Crackers** | *Available* | 80 | n/a |  | 26.3 | 36.3 | 36.3 |
|  | *Overall* | 71 | 203.3 |  | 6.8 | 7.9 | 7.9 |
|  | *Children* | 54 | 49.3 |  | 2.9 | 3.6 | 3.6 |
|  | *Adults* | 70 | 154.0 |  | 8.1 | 9.2 | 9.2 |
| **Snack/meal bars** | *Available* | 46 | n/a |  | 0 | 2.2 | 6.5 |
|  | *Overall* | 44 | 92.6 |  | 0 | 0.7 | 7.3 |
|  | *Children* | 38 | 21.8 |  | 0 | 0.1 | 7.4 |
|  | *Adults* | 42 | 70.8 |  | 0 | 0.9 | 7.3 |
| **Smoothies and grain drinks** | *Available* | 19 | n/a |  | 47.4 | 63.2 | 63.2 |
|  | *Overall* | 19 | 42.7 |  | 78.4 | 88.4 | 88.4 |
|  | *Children* | 18 | 7.3 |  | 63.5 | 80.8 | 80.8 |
|  | *Adults* | 19 | 35.3 |  | 81.5 | 89.9 | 89.9 |
| **Baby food: cereals, snacks, sweets** | *Available* | 55 | n/a |  | 80.0 | 78.2 | 80.0 |
|  | *Overall* | 13 | 1.03 |  | 65.5 | 65.5 | 65.5 |
|  | *Children* | 13 | 1.00 |  | 64.6 | 64.6 | 64.6 |
|  | *Adults* | 2 | 0.03 |  | 100 | 100 | 100 |
| **Mixed dishes** | *Available* | 619 | n/a |  | 62.7 | 72.4 | 77.4 |
|  | *Overall* | 471 | 422.8 |  | 54.7 | 70.8 | 67.5 |
|  | *Children* | 339 | 117.3 |  | 46.8 | 67.6 | 58.8 |
|  | *Adults* | 425 | 305.5 |  | 57.7 | 72.0 | 70.8 |
| **Total** | *Available* | 2208 | n/a |  | 44.0 | 51.4 | 52.9 |
|  | *Overall* | 1736 | 3312.0 |  | 44.1 | 51.4 | 51.7 |
|  | *Children* | 1330 | 862.6 |  | 35.9 | 43.7 | 42.5 |
|  | *Adults* | 1621 | 2449.4 |  | 47.0 | 54.1 | 54.9 |

^a^ We used data from the two most recent National Health and Nutrition Examination Survey (NHANES) cycles (2013-14, 2015-16) to identify carbohydrate (carb)-rich products consumed by Americans. Products were aggregated into 12 food categories based on the WWEIA food categories. The mixed dish category included rice mixed dishes, pasta mixed dishes, macaroni and cheese, turnovers and other grain-based items, fried-risk and lo/chow mein. Additional mixed dishes including (e.g., meat mixed dishes, sandwiches, soups, pizza, burgers, etc.) were included if they contained ≥ 50% of energy from carbohydrates and ≥0.25 ounce-equivalents of total grains.

^b^ Children included individuals aged 2-19 years old and adults included individuals aged 20 years and older.

^c^ Frequency means that products were weighted by their actual reported consumption levels overall or by children or adults, with products consumed more frequently receiving greater weight.

^d^ Ofcom model was developed for the regulation of television advertising to children in the United Kingdom. Foods with a final score of <4 points and beverages scoring <1 point are considered as healthy. The FSANZ (Food Standards Australia New Zealand) model was developed for the regulation of health claims on foods in Australia and New Zealand. Foods with a final score of <4 points and beverages scoring <1 point are meeting score criteria to carry a health claim. The HSR (Health Star Rating) is a government led initiative that scores the nutritional value of packaged foods. It is designed to help consumers make healthier choices when shopping within a category. The score of HSR ranges from ½ star to 5 stars. Foods and beverages with a final score of 3.5 points or more is considered as green or nutritious.

# **Table S16. Comparisons of available carbohydrate-rich projects meeting each of three nutrient profiling systems by meeting or not meeting each four proposed metrics**

| **Four metrics^a^** | **Analysis^b^** | **Products** | |  | **% Meeting Nutrient Profiling Systems^d^** | | |
| --- | --- | --- | --- | --- | --- | --- | --- |
|  |  | **#** | **Frequency^c^**  **(million)** |  | **Ofcom**  **(Healthy)** | **FSANZ**  **(Health claim)** | **HSR**  **(Green or nutritious)** |
| **10:1** |  |  |  |  |  |  |  |
| Yes | *Available* | 512 | n/a |  | 64.1 | 76.6 | 80.5 |
| No | *Available* | 1696 | n/a |  | 38.0 | 43.9 | 44.5 |
| **10:1:1** |  |  |  |  |  |  |  |
| Yes | *Available* | 362 | n/a |  | 73.5 | 82.9 | 84.5 |
| No | *Available* | 1846 | n/a |  | 38.2 | 45.3 | 46.6 |
| **10:1:2** |  |  |  |  |  |  |  |
| Yes | *Available* | 423 | n/a |  | 70.2 | 82.3 | 84.2 |
| No | *Available* | 1785 | n/a |  | 37.8 | 44.1 | 45.4 |
| **10:1\|1:2** |  |  |  |  |  |  |  |
| Yes | *Available* | 471 | n/a |  | 66.9 | 79.6 | 83.9 |
| No | *Available* | 1737 | n/a |  | 37.8 | 43.8 | 44.4 |

**^a^**We defined and applied four different carb metrics: per 10g of carb:(a) ≥1g fiber (10:1 carb:fiber), (b) ≥1g fiber and <1g free sugars (10:1:1 carb:fiber:free sugars), (c) ≥1g fiber and <2g free sugars (10:1:2 carb:fiber:free sugars); and (d) ≥1g fiber and, per each 1 g of fiber, <2g free sugars (10:1 carb:fiber, 1:2 fiber:free sugars; or 10:1|1:2).

^b^ Available refers to the existing products at the Food and Nutrient Database for Dietary Studies.

^c^Frequency means that products were weighted by their actual reported consumption levels overall or by children or adults, with products consumed more frequently receiving greater weight.

^d^ Ofcom model was developed for the regulation of television advertising to children in the United Kingdom. Foods with a final score of <4 points and beverages scoring <1 point are considered as healthy. The FSANZ (Food Standards Australia New Zealand) model was developed for the regulation of health claims on foods in Australia and New Zealand. Foods with a final score of <4 points and beverages scoring <1 point are meeting score criteria to carry a health claim. The HSR (Health Star Rating) is a government led initiative that scores the nutritional value of packaged foods. It is designed to help consumers make healthier choices when shopping within a category. The score of HSR ranges from ½ star to 5 stars. Foods and beverages with a final score of 3.5 points or more is considered as green or nutritious.

Supplemental Figure 1. Food items flow chart

Food codes

FNDDS 2013-2014

FNDDS 2015-2016

FNDDS version by NHANES survey cycle

N=8,536

N=8,690

N=9,514

Combined total food codes

N=2,208

Included food codes, i.e. carbohydrate-rich food products for the analysis

**References**

1. World Health Organization. Nutrient profiling. <https://www.who.int/nutrition/topics/profiling/en/>. Accessed on May 28, 2019.

2. Labonte ME, Poon T, Gladanac B, Ahmed M, Franco-Arellano B, Rayner M and L'Abbe MR. Nutrient Profile Models with Applications in Government-Led Nutrition Policies Aimed at Health Promotion and Noncommunicable Disease Prevention: A Systematic Review. *Adv Nutr*. 2018;9:741-788.

3. Rayner M, Scarborough P and Lobstein T. The UK Ofcom Nutrient Profiling Model: Defining ‘healthy’and ‘unhealthy’foods and drinks for TV advertising to children. *London: OfCom*. 2009.

4. UK Department of Health and Social Care. The nutrient profiling technical guidance. Available at : <https://assets.publishing.service.gov.uk/government/uploads/system/uploads/attachment_data/file/216094/dh_123492.pdf>. Accessed on May 31, 2019.

5. Food Standards Australia New Zealand. Available at <http://www.foodstandards.gov.au/industry/labelling/Pages/Short-guide-for-industry-to-the-NPSC.aspx>. Accessed on May 31, 2019.

6. Ni Mhurchu C, Brown R, Jiang Y, Eyles H, Dunford E and Neal B. Nutrient profile of 23 596 packaged supermarket foods and non-alcoholic beverages in Australia and New Zealand. *Public Health Nutr*. 2016;19:401-8.

7. Australia and New Zealand Food Regulation Secretariat. Front-of-Pack Labelling Committee andWorking

Group Meetings. Available online: <http://foodregulation.gov.au/internet/fr/publishing.nsf/Content/>

frontofpackcommittee. Accessed on May 31, 2019.

8. Health Star Rating Advisory Committee (HSRAC), Department of Health. Guide for Industry to the

Health Star Rating Calculator. Available online: <http://healthstarrating.gov.au/internet/healthstarrating/>

publishing.nsf/content/guide-for-industry-document. Accessed on May 28, 2019.
